# Evaluation of Red Blood Cell Biochemical Markers and Coagulation Profiles Following Cell Salvage in Cardiac Surgery: A Systematic Review and Meta-Analysis

**DOI:** 10.3390/jcm13206073

**Published:** 2024-10-11

**Authors:** Rocío Cáceres-Matos, Manuel Luque-Oliveros, Manuel Pabón-Carrasco

**Affiliations:** 1 Research Group PAIDI-CTS-1050, “Complex Care, Chronicity and Health Outcomes”, Faculty of Nursing, Physiotherapy and Podiatry, University of Seville, 41009 Seville, Spain; rcaceres3@us.es; 2 Cardiovascular and Thoracic Surgery Operating Theatre Unit, Faculty of Nursing, Physiotherapy and Podiatry, Virgen Macarena University Hospital, University of Seville, 41009 Seville, Spain

**Keywords:** biochemical markers, red blood cell, blood coagulation, surgical blood loss, operative blood salvage, blood transfusion autologous, blood transfusion

## Abstract

**Background**: Individuals undergoing cardiac surgery face an increased risk of bleeding, as well as alterations in biochemical and coagulation patterns. Therefore, assessing the effectiveness of systems such as Cell Salvage is necessary to prevent potential surgical complications. **Objective**: To evaluate the efficacy of Cell Salvage in relation to the biochemical parameters of the red blood series and coagulation, as well as the risk of hemorrhage. **Methods**: A systematic review, accompanied by a meta-analysis, was executed via an extensive literature exploration encompassing Medline, CINAHL, Scopus, Web of Science, and the Cochrane Library. The inclusion criteria comprised studies in English or Spanish, without year restrictions, conducted in adults and with a randomized controlled trial design. **Results**: Twenty-six studies were included in the systematic review, involving a total of 2850 patients (experimental group = 1415; control group = 1435). Cell Salvage did not demonstrate superior outcomes compared to allogeneic transfusions in the management of post-surgical hemorrhage, as well as in total blood loss, platelet count, fresh frozen plasma, and fibrinogen. However, Cell Salvage showed a greater effectiveness for hemoglobin (moderate evidence), hematocrit (low evidence), post intervention D-dimer (low evidence), and some coagulation-related parameters (low evidence) compared to allogeneic transfusions. Finally, better results were found in the control group for INR parameters. **Conclusions**: The use of the Cell Salvage system holds high potential to improve the postoperative levels of biochemical and coagulation parameters. However, the results do not provide definitive evidence regarding its effectiveness for hemorrhage control, platelet count, fresh frozen plasma, and fibrinogen. Therefore, it is recommended to increase the number of studies to assess the impact of the Cell Salvage system on improvements in the red blood cell count and patient coagulation patterns. In addition, protocols should be homogenized, and variables such as the sex of the participants should be taken into account.

## 1. Introduction

Throughout history, cardiac surgery has been a major consumer of blood products worldwide, and specifically, it is the surgery that consumes the most blood resources [1,2,3]. Patients undergoing cardiac surgery face a high risk of bleeding [4,5]. Consequently, these patients receive a significant amount of allogeneic blood transfusions, accounting for between 15% and 20% of perioperative transfusions [6,7]. Similarly, in recent years, there has been a decline in blood donations, a trend exacerbated by the COVID-19 pandemic [8,9,10].

On the other hand, there is concern about the side effects of allogeneic blood transfusion. This has driven the development of methods aimed at minimizing these risks in the perioperative period. These methods include preoperative autologous donation (PAD) [11,12], isovolemic hemodilution [13], and the use of medications such as aprotinin [14], tranexamic acid [15], and erythropoietin [16].

In addition to the mentioned methods, there is the Cell Salvage approach, which can reduce the need for allogeneic blood transfusions during the perioperative period. Cell Salvage is well-established in cardiac surgery [17]. It allows for the reinfusion of blood extracted from the surgical field, either directly or after a centrifugation process to remove non-cellular components [18]. This potentially reduces the risk of blood transfusions while maintaining acceptable hemoglobin (Hb) levels during the intraoperative and early postoperative periods [19,20]. In addition to its primary role in managing patient blood [21], there is evidence that Cell Salvage is associated with a decrease in systemic inflammation and a reduced incidence of postoperative atrial fibrillation, a common arrhythmia after cardiac surgery [22]. The first autotransfusion of blood extracted from a patient was described in 1860 [23]. The devices used at that time were often associated with serious complications, such as gas embolism, although nowadays, these complications are rare [24].

While numerous articles have been published on Cell Salvage use, there is considerable controversy regarding its effectiveness, with studies supporting its use [25,26,27] and others opposing it due to a lack of conclusive results [28,29].

Due to the variability in methodologies, procedures, and results obtained, there is a need for a synthesis of the existing literature to analyze the effectiveness of Cell Salvage and update the latest findings. Furthermore, exploring alternative approaches to allogeneic transfusions becomes more necessary when facing a lack of blood resources in blood banks, along with the side effects and the high prevalence of bleeding in cardiac surgery. Therefore, the objective of this meta-analysis of randomized clinical trials was to evaluate the effectiveness of Cell Salvage regarding biochemical parameters of the red blood cell series in the complete blood count and coagulation, as well as the risk of bleeding.

## 2. Materials and Methods

### 2.1. Search Strategy and Inclusion Criteria

An exhaustive search was conducted in databases such as Medline (PubMed), CINAHL, Scopus, Web of Science, and The Cochrane Library, following the recommended guidelines in Preferred Reporting Items for Systematic Reviews and Meta-Analyses for Protocols (PRISMA) [30]. This search was assessed using A Measurement Tool to Assess Systematic Reviews (AMSTAR-2) [31,32]. Additionally, a search was carried out on ClinicalTrials.gov, confirming the absence of similar documents in the Prospero registry. Google Scholar was also explored to identify unconventional literature and minimize potential publication biases. The protocol for this study was registered on the Prospero website in July 2023 (CRD42023446583).

A PICO question was formulated as follows: Patient/Problem: Individuals undergoing cardiovascular, cardiac, and/or thoracic surgery; Intervention: Use of a cell saver; Comparison: Conventional blood transfusion or alternative controls; Outcomes: Evaluation of the reduction in bleeding risk, along with key biochemical parameters, including hemoglobin levels, platelet count, prothrombin time, and activated partial thromboplastin time.

The search terms used were (“blood retrievers” OR “intraoperative cell salvage” OR “autotransfusion system” OR “cell saver” OR “Operative Blood Salvage” OR “Blood Transfusion, Autologous”) AND (“cardiac surgery” OR “cardiopulmonary bypass”) AND (“Hemoglobins” OR “Hemorrhage” OR “Hematocrit” OR “Erythrocyte Count” OR Erythrocytes OR “Blood Component Transfusion” OR “International Normalized Ratio” OR “Partial Thromboplastin Time” OR “Prothrombin Time” OR “Thrombin Time” OR “fresh frozen plasma” OR “Platelet Count” OR “Pyrimidine Dimers” OR “Fibrinogen”) and their equivalent in Spanish or French. The search equation descriptors were chosen from the Medical Subject Headings (MeSH) thesaurus.

These search terms were obtained from the Medical Subject Headings (MeSH) and were used during the search conducted from January to June 2024 by two researchers (M.P.-C. and R.C.-M.). The inclusion criteria comprised articles published without year restriction, in English or Spanish, related to the objectives of this study, and randomized clinical trials (RCTs) conducted in adult humans. The exclusive selection of RCTs was performed to enhance the methodological quality of the review and mitigate biases in an intrinsically complex subject due to the diversity of factors influencing its success, such as the type of surgery, cardiopulmonary bypass time, professional experience, patients’ body mass index, and patients’ comorbidities, among others.

### 2.2. Data Extraction

The search and article selection were independently conducted by two researchers (M.P.-C. and R.C.-M.), and in the case of disagreement, the opinion of an expert in cardiac surgery was sought for resolution. Initially, the titles and abstracts of articles were reviewed, followed by a full article assessment. Additionally, a bibliographic search was performed both forward and backward in the references cited in the selected studies. The agreement between the two researchers in assessing the suitability of the studies was quantified using the Kappa statistical test.

A data coding manual was followed to gather information from each study, including (1) author’s name; (2) year of publication; (3) country of origin; (4) study design; (5) sample size; (6) type of intervention (use of Cell Salvage versus control group); (7) participants’ age; (8) objectives of each study; and (9) outcomes obtained. The primary dichotomous outcomes analyzed included continuous outcomes focused on hemorrhage, FFP, hemoglobin, fibrinogen, aPTT and aPTT ratio, PT, TT, D-dimer, and INR.

### 2.3. Quality and Bias Risk Assessment

The Cochrane Risk of Bias Tool [33] was utilized, categorizing each type of risk into three levels: low, high, or unclear. The evaluated risk types encompassed aspects such as random sequence generation, allocation concealment, blinding of participants and personnel, blinding in outcome assessment, integrity of outcome data, selective reporting, and other potential sources of bias. Studies without a high risk of bias in any category were considered high quality (1++), while those with a high risk or two unclear risks were rated as medium quality (1+). Other studies were considered low quality (1−).

Additionally, the modified Jadad scale was applied to assess the internal validity of each study, where a score of ≥4 indicated high quality.

For the risk of bias assessment, the Cochrane Handbook for Intervention Reviews (Revman Version 5.4) was used. Two independent reviewers subjectively assessed all articles and assigned ratings of “high”, “low”, or “unclear” based on selection, performance, detection, attrition biases, and other potential biases. Disagreements were resolved through discussions to reach a consensus. If a consensus was not reached, the opinion of a third investigator (M.-L.O.) was sought.

Statistical analysis and bias assessment were conducted using the Review Manager software, version 5.4 (Cochrane Library, London, UK). Additionally, the data were imported into the Grade Pro application, allowing for the assessment of the recommendation grade for the obtained data [34].

### 2.4. Data Synthesis and Statistical Analysis

The relative risk (RR) was used to compare dichotomous variables, and 95% confidence intervals (CIs) were provided. Continuous variables were evaluated using mean differences (MDs) along with a 95% CI. Data for dichotomous outcomes were pooled using a random-effects model [6] to provide a more cautious estimate of the effects of Cell Salvage. When standard deviation data were not available in the study, the method recommended by Hozo et al. was applied [35]. Both binary and continuous data were calculated using fixed or random-effects models. The fixed-effects model was chosen initially if there was no significant heterogeneity between studies (I^2^ ≤ 50%). Otherwise, the random-effects model was used [36].

Heterogeneity among studies was assessed through chi-square tests and the I^2^ test, with a statistical significance level of *p*-value < 0.05. I^2^ values between 0% and 25% indicated low heterogeneity, between 25% and 75% moderate heterogeneity, and over 75% high heterogeneity [37].

A forest plot was used to visualize the results of the meta-analysis, and a funnel plot was employed to assess possible publication bias among studies. The asymmetry of the funnel plot was analyzed using the funnel plot representation and evaluated with the Egger’s test, considering a statistical significance level of *p*-value < 0.05 as indicative of publication bias evidence.

Subgroup analysis based on biochemical patterns was conducted. A sensitivity analysis was also performed to assess the robustness of the results by sequentially omitting each study. *p*-values < 0.05 were considered statistically significant.

The comparison of the impact of Cell Salvage in relation to allogeneic transfusions was expressed as the RR of reintervention or subsequent bleeding, along with 95% confidence intervals. The units of measurement in which the variables were expressed were as follows: for bleeding and the volume of FFP in milliliters (mL); hemoglobin and fibrinogen in grams per deciliter (g/dL); aPTT, PT, and TT in seconds; and D-dimer in nanograms per milliliter (ng/mL).

## 3. Results

### 3.1. Results Obtained in the Selection of Articles

**Figure 1:**
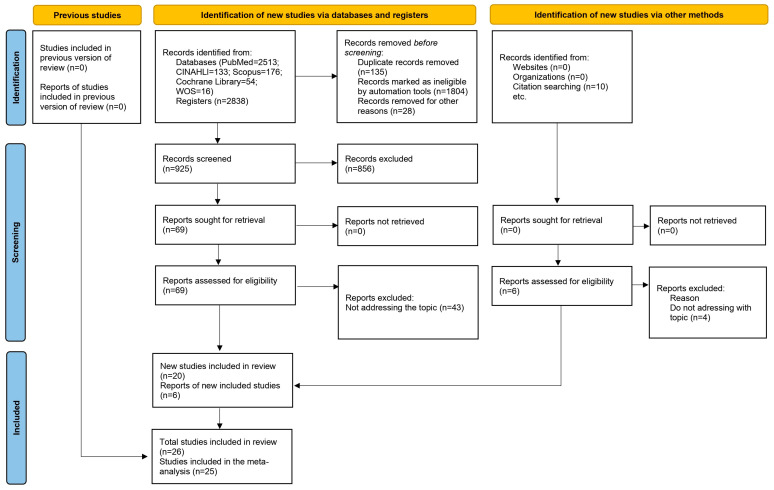
Flow diagram that illustrates the review process.

In the initial literature search, a total of 2838 articles were identified, with no additional documents excluded from specific clinical trial registries (ClinicalTrials.gov and Prospero). After removing 135 duplicate articles using the Zotero^®^ reference manager, applying the inclusion criteria, and evaluating the titles and abstracts of the articles, 856 were excluded for not meeting the inclusion criteria. Finally, 26 studies were selected for the systematic review analysis, of which 25 provided data for the meta-analysis, encompassing a sample of 2850 participants (74.4% men vs. 25.6% women) who underwent cardiac surgery (experimental group with Cell Salvage, *n* = 1415; control group, *n* = 1435). The flow diagram (Figure 1) illustrates the review process. There was excellent agreement between the researchers regarding the eligibility assessment of the trials (Kappa statistic = 0.95).

### 3.2. Descriptive Analysis of the Results Found

The years with the highest scientific production were 2015, with three articles, and 2006, 2007, and 2012, each with two publications. The levels of evidence assessed based on the quality of the selected articles received a score of 1++ in 13.63% (*n* = 3) of cases, 27.27% received a score of 1+ (*n* = 6), and 59.09% received a score of 1− (*n* = 13).

The included studies addressed hemorrhage (*n* = 11; 42.31%), hemoglobin (*n* = 18; 69.23%), hematocrit (*n* = 7; 26.92%), fibrinogen (*n* = 4; 15.38%), INR (*n* = 2; 7.69%), aPTT (*n* = 5; 19.23%), PT (*n* = 3; 11.54%), TT (*n* = 1; 3.85%), platelet count (*n* = 11; 42.31%), and aPTT ratio (*n* = 2; 7.69%). The details of each item included are provided in Table 1.

A moderate grade of recommendation was observed for overall hemoglobin (MD 0.48, 95 CI 0.28–0.69), immediate postoperative hemoglobin (MD 0.65, 95 CI 0.27–1.04), and hemoglobin at 24 h (MD 0.56, 95 CI 0.23–0.90). All other variables had a low or very low grade of recommendation. All analyses were performed using Grade PRO^®^ (Table S1).

### 3.3. Bias Risk Assessment of the Selected Studies and Publication Bias

The risk of bias was assessed using RevMan 5^®^, represented in Supplementary Figures S1 and S2 by the bias assessment plots of all included studies and by a one-to-one summary plot. Allocation concealment was evident in about 35% of included studies, with approximately 15% blinding of participants and staff and 20% blinding of outcome assessment. In relation to publication bias, a funnel plot for each study objective assessed shows an inverted funnel, with the strongest studies concentrated in the center (Figure S3).

### 3.4. Results of the Meta-Analysis

#### 3.4.1. Efficacy of Cell Salvage in Hemorrhage

In eleven clinical trials involving 2056 participants, with 1015 in the intervention group and 1041 in the control group, the efficacy of utilizing Cell Salvage in hemorrhage was assessed [38,43,45,54,55,56,57,59,60,61,63]. Seven studies exhibited a high risk of bias [38,43,57,59,60,61,63].

At six hours post-surgical intervention, blood losses were higher in the control group than in the Cell Salvage group in three studies. However, no significant differences were found between the two groups. An MD of 11.92 was obtained, with a 95% confidence interval of −80.21 to 104.04 (*p* = 0.80), and significant heterogeneity was noted among the studies (I^2^ = 84%, *p* < 0.001).

Twelve hours post-surgical intervention, both studies hit the no-effect line with no difference found between the two groups. An MD of 25.71 was obtained, with a 95% confidence interval of −64.16 to 115.58 (*p* = 0.57), and heterogeneity was found among the studies (I^2^ = 0%, *p* = 0.45).

Regarding the amount of blood lost at 24 h, four studies tended to have higher losses in the control group, two in the intervention group, and another two had similar losses between the two groups. We obtained an MD of 0.79, with a 95% confidence interval of −67.07 to 68.65 (*p* = 0.98), and heterogeneity between studies (I^2^ = 59%, *p* = 0.02).

Concerning the total amount of blood lost, one study found greater losses in the control group and another in the intervention group. An MD of 217.92 was obtained, with a 95% confidence interval of −489.53 to 925.37 (*p* = 0.55), and significant heterogeneity among the studies (I^2^ = 98%, *p* < 0.001).

**Figure 2:**
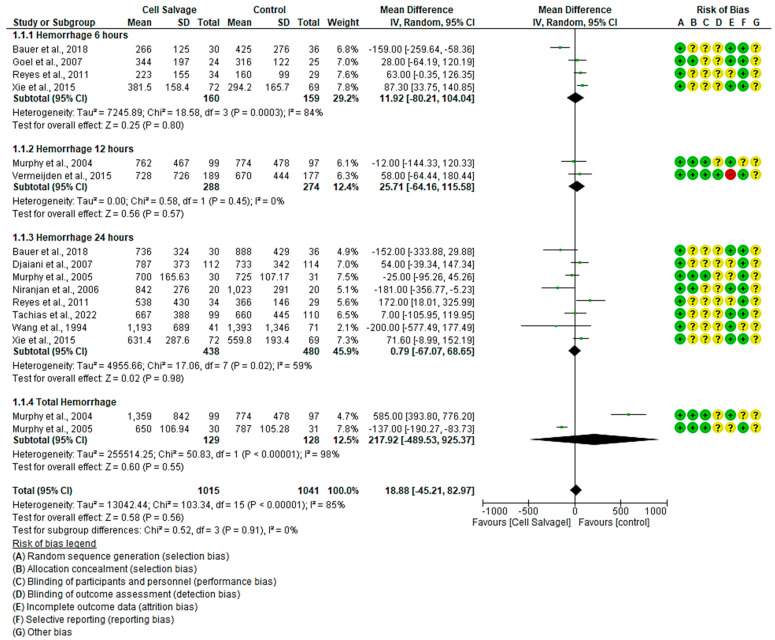
Forest plot depicting hemorrhage at different time slices.

Ultimately, no statistically significant differences were observed between the experimental and control group (*p* = 0.56). This is evident in the confidence intervals as well as in the forest plot (no-effect line) (Figure 2).

#### 3.4.2. Efficacy of Cell Salvage on Hemoglobin Levels

In seventeen clinical trials involving 3671 participants, with 1806 in the intervention group and 1865 in the control group, the efficacy of utilizing Cell Salvage on hemoglobin was examined. Eleven studies exhibited a high risk of bias [38,41,43,46,49,52,57,58,59,60,61,62,63].

In the immediate postoperative period, hemoglobin levels were higher in eight studies, while one favored Cell Salvage. In three studies, the results were not statistically significant, approaching the no-effect line. An MD of 0.65 was obtained, with a 95% confidence interval of 0.27 to 1.04 (*p* < 0.001), and there was significant heterogeneity among the studies (I^2^ = 91%, *p* < 0.001).

At six hours, more ambiguous results were shown: two studies tended to have better hemoglobin results in the Cell Salvage group, while one study favored the control group. An MD of 0.39, with a 95% confidence interval of −0.21 to 1.00 (*p* = 0.20), and heterogeneity between studies (I^2^ = 80%, *p* < 0.05) were obtained, so that at 6 h, no significant differences between groups were obtained.

After twenty-four hours of surgery, a total of ten clinical trials were found. The results show an improvement in Cell Salvage compared to the control group. The MD was 0.46, with a 95% confidence interval of 0.23 to 0.90 (*p* = 0.0001), and significant heterogeneity was noted between studies (I^2^ = 92%, *p* < 0.001).

Regarding the amount of hemoglobin at discharge, four studies did not show statistically significant differences, approaching the no-effect line. An MD of −0.05 was obtained, with a 95% confidence interval of −0.23 to 0.13 (*p* = 0.57), and no heterogeneity among the studies was observed (I^2^ = 0%, *p* = 0.77).

**Figure 3:**
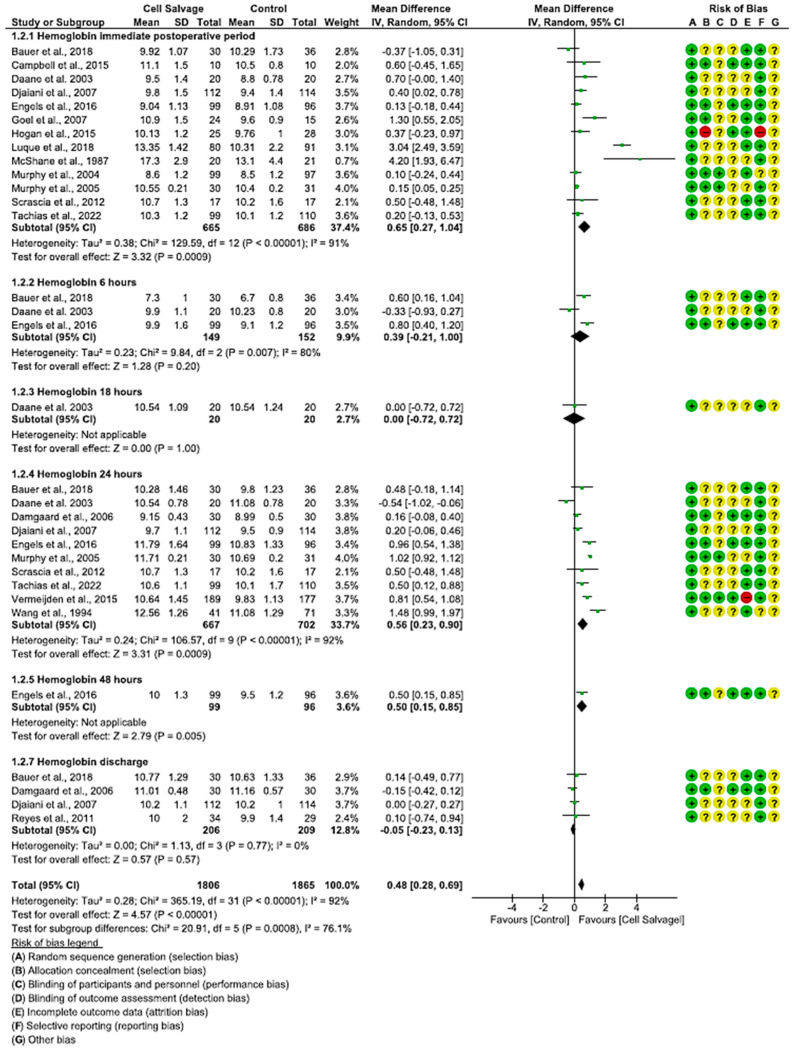
Forest plot presenting hemoglobin at different time slices.

Finally, statistically significant differences were observed (*p* < 0.001), with the Cell Salvage being more efficient (Figure 3).

#### 3.4.3. Efficacy of Cell Salvage on Hematocrit

In six clinical trials involving 666 participants, with 311 in the intervention group and 355 in the control group, the efficacy of using Cell Salvage to improve hematocrit was compared with traditional methods based on transfusions. Five studies showed a high risk of bias [38,41,42,43,45], while one exhibited an adequate quality level [42].

In the immediate postoperative period, four studies indicated better scores for the Cell Salvage group, while only one did not show a statistically significant association, approaching the no-effect line. The results showed an MD of 3.91, with a 95% confidence interval of 0.44 to 7.37 (*p* = 0.03), and significant heterogeneity between studies (I^2^ = 99%, *p* < 0.001). Therefore, an improvement in hematocrit was observed in the Cell Salvage group.

There was only one study that assessed hematocrit both at 6 h and at 18 h. The results tended to favor the Cell Salvage (*p* = 0.29).

At 24 h, while two studies showed better levels for the Cell Salvage group, one favored the control group. An MD of 2.33 was obtained, with a 95% confidence interval of −0.07 to 4.74 (*p* = 0.06), and there was significant heterogeneity between studies (I^2^ = 94%, *p* < 0.001).

**Figure 4:**
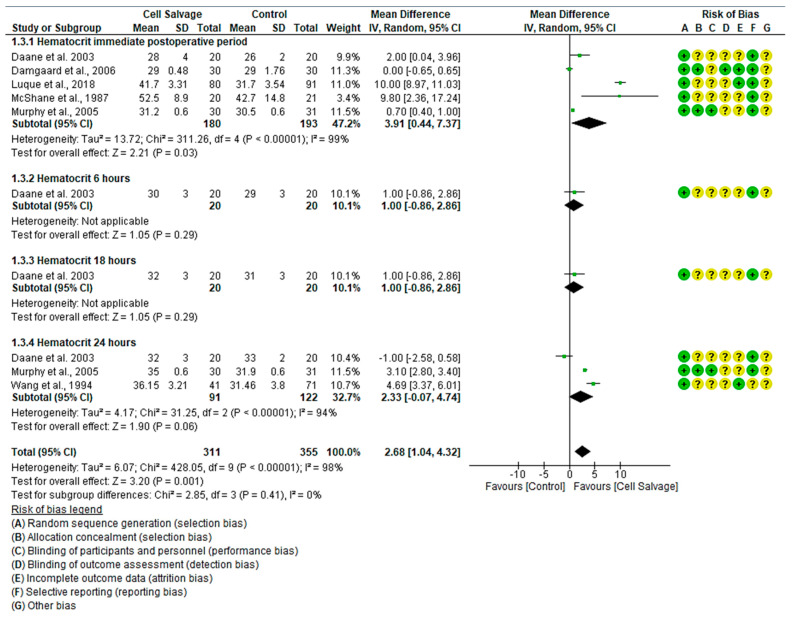
Forest plot presenting hematocrit at different time slices.

Finally, statistically significant differences were observed in favor of the Cell Salvage (*p* < 0.001) (Figure 4).

#### 3.4.4. Efficacy of Cell Salvage on Coagulation Parameters

For aPTT, five clinical trials with 1363 participants were identified, with 665 in the intervention group and 698 in the control group, comparing the efficacy of Cell Salvage use. All five studies showed a high risk of bias [39,41,43,46,59]. In the immediate postoperative period, two studies indicated better aPTT times for the control group, while one clearly favored the use of Cell Salvage. The rest have disparate results based on their confidence intervals. An MD of 0.87 was obtained, with a 95% confidence interval of −5.01 to 6.76 (*p* = 0.77), and significant heterogeneity was observed among the studies (I^2^ = 97%, *p* < 0.001).

At 6 h (*p* = 0.21) and 18 h (*p* = 0.43) post-surgical intervention, and upon hospital discharge (*p* = 0.93), aPTT was only assessed in one study, showing a trend toward better times for Cell Salvage.

At 24 h, three studies showed no difference between the groups. It can be seen that the confidence intervals touch the no-effect line. An MD of 0.77, with a 95% confidence interval of −0.46 to 2.00 (*p* = 0.22) was obtained, and heterogeneity was noted between studies (I^2^ = 48%, *p* < 0.001).

Finally, no statistically significant differences were found between groups (95% CI = −2.50 to 3.49, *p* = 0.74).

For the aPTT ratio, two clinical trials with 575 participants, 288 in the intervention group and 287 in the control group, were identified, comparing the efficacy of Cell Salvage use. Both studies showed a high risk of bias [55,56]. In the immediate postoperative period, both studies indicated better aPTT ratio values for the intervention group with Cell Salvage, with an MD of −0.05, a 95% confidence interval of −0.06 to −0.04 (*p* < 0.001), and no heterogeneity among the studies (I2 = 0%, *p* = 0.33). At 24 h post-surgical intervention, only one study evaluated the aPTT ratio, finding no statistically significant differences between groups, approaching the no-effect line. Lastly, statistically significant differences were found between groups (*p*-value < 0.001).

**Figure 5:**
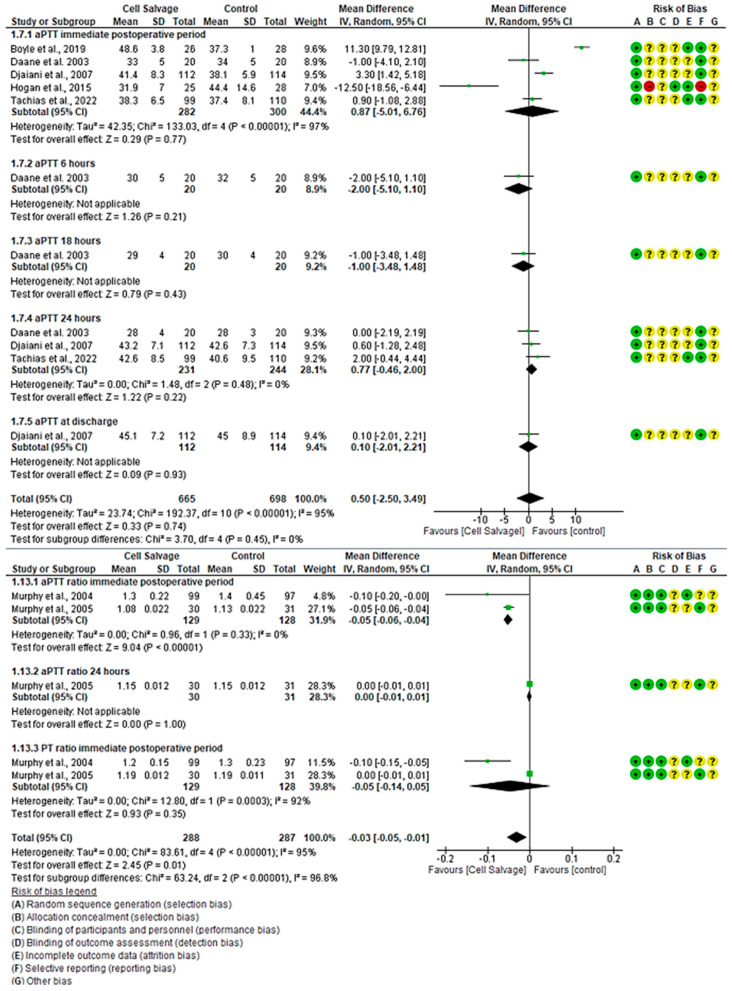
Forest plot presenting the aPPT and aPPT ratio over time.

Regarding the PT ratio, two clinical trials with a total of 257 participants, 129 in the intervention group and 128 in the control group, were identified, both showing a medium risk of bias [54,55]. One of the two clinical trials showed better values for Cell Salvage, while the second did not show a statistically significant association, approaching the no-effect line. An MD of −0.05 was obtained, with a 95% confidence interval of −0.14 to 0.05 (*p* = 0.35), and significant heterogeneity among the studies was observed (I^2^ = 92%, *p* < 0.001). Results favoring Cell Salvage are evident (Figure 5).

For PT, three clinical trials with 267 participants were identified, with 131 in the intervention group and 136 in the control group, comparing the efficacy of Cell Salvage use with conventional techniques based on traditional transfusions. All three studies showed a high risk of bias [39,43,46]. Regarding PT, two studies demonstrated better results for the Cell Salvage, while one did not show a statistically significant relationship, touching the no-effect line. An MD of −0.88 was obtained, with a 95% confidence interval of −1.48 to −0.29 (*p* < 0.001), with heterogeneity among the studies (I^2^ = 66%, *p* = 0.05). At 6, 18, and 24 h, only one study evaluated PT, finding no statistically significant association, touching the no-effect line (*p* = 0.999). Finally, there was also no association between groups when assessing combined outcomes, touching the no-effect line (*p* = 0.16).

**Figure 6:**
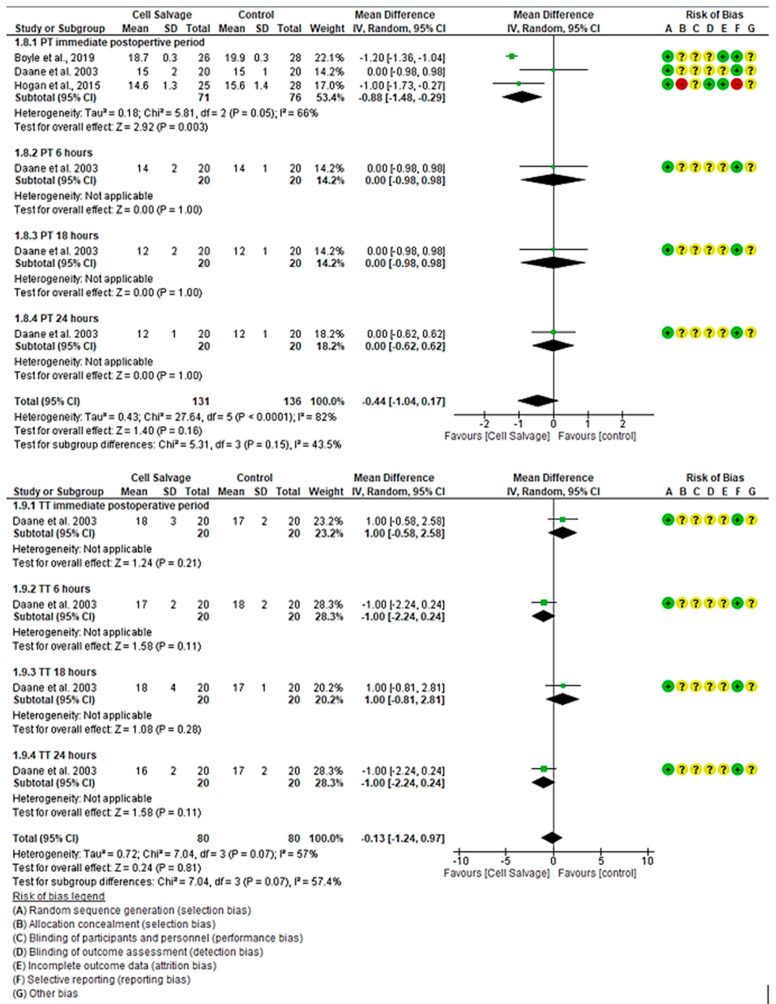
Forest plot presenting PT and TT over time.

For TT, one clinical trial with 160 participants was identified, with 80 in the intervention group and 80 in the control group, comparing the efficacy of Cell Salvage use with traditional transfusions. The study showed a high risk of bias [43]. In the immediate postoperative period and at 18 h, the study demonstrated better results for the control group, while it was better in the Cell Salvage group at 6 and 24 h. However, all confidence intervals run along the no-effect line (Figure 6).

#### 3.4.5. The Efficacy of Cell Salvage in Fresh Frozen Plasma Transfusion

In eleven clinical trials, comprising a total of 1153 participants in both the intervention group (*n* = 602) and the control group (*n* = 551), the effectiveness of using Cell Salvage was compared with the control group, where traditional methods based on allogeneic transfusion were employed. Seven studies exhibited a high risk of bias [43,45,50,53,57,58,63]. Regarding the number of individuals transfused with FFP, a total of 100 transfusion cases (16.61%) were observed in the intervention group, while this figure rose to 72 (13.07%) in the control group. One study indicated a higher number of individuals transfused in the control group, while another study showed more cases for the intervention group. The remaining studies yielded inconclusive results, with confidence intervals bordering on the no-effect line. No statistically significant differences were found between the two groups (OR 1.28, 95% CI = 0.92 to 1.78), with moderate heterogeneity between studies (I^2^ = 44%, *p* = 0.06).

For platelet count, a total of 2186 participants were involved in eleven clinical trials, with 1062 in the intervention group and 1126 in the control group, comparing the efficacy of Cell Salvage use. Seven studies exhibited a high risk of bias [41,43,46,49,57,59,61], while the remaining studies showed low levels of bias [40,44,54,55]. In the immediate postoperative period, one study indicated a higher number of individuals transfused in the control group, while another study showed more cases for the intervention group. The remaining studies yielded inconclusive results, with confidence intervals bordering on the no-effect line to a greater or lesser extent. An MD of −0.24 was obtained, with a 95% confidence interval of −4.71 to 4.23 (*p* = 0.92), and there was significant heterogeneity among the studies (I^2^ = 54%, *p* < 0.02).

At 24 h, among the four studies that evaluated platelet count, only one study showed better results for the group that used Cell Salvage. An MD of 0.11 was obtained, with a 95% confidence interval of −8.76 to 8.98 (*p* = 0.98), and there was significant heterogeneity between studies (I^2^ = 71%, *p* < 0.001).

No group differences were found at both 6 h (*p* = 0.10) and 18 h (*p* = 0.73). Likewise, there was no group difference at patient discharge (*p* = 0.24). In these cases, only one study was found.

**Figure 7:**
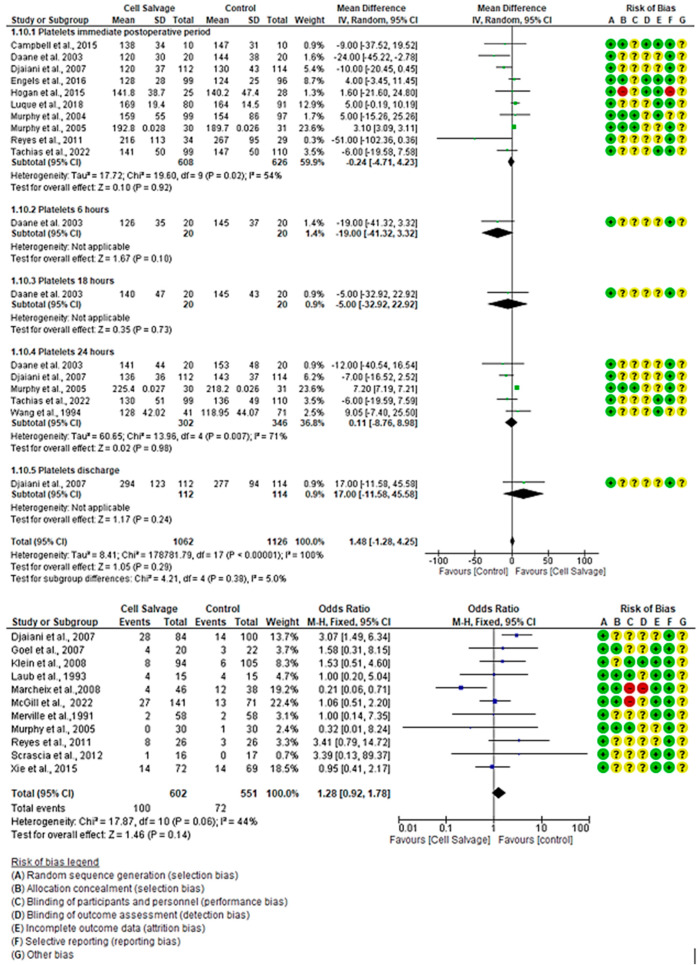
Forest plot presenting platelet count over time and number of fresh frozen plasma transfusions.

Finally, no statistically significant differences were found between groups (*p*-value = 0.29) (Figure 7).

#### 3.4.6. The Efficacy of Cell Salvage on Other Coagulation Factors

Four clinical trials assessed postoperative fibrinogen levels with a total of 362 participants, including 172 in the intervention group and 189 in the control group, comparing the efficacy of Cell Salvage use. Four studies demonstrated a high risk of bias [39,41,59], while the remaining study showed a low risk of bias [55]. In the immediate postoperative period, one study showed better levels in the group that employed Cell Salvage and another in the control group. The remaining two studies did not show a statistically significant association, with the diamond touching the no-effect line. An MD of 0.11 was obtained, with a 95% confidence interval of −0.07 to 0.29 (*p* = 0.23), and there was high heterogeneity among the studies (I^2^ = 96%, *p* < 0.001).

At 24 h, among the three studies assessing fibrinogen levels, one study showed better levels for the control group, while the other two invaded the no-effect line. An MD of −0.01 was obtained, with a 95% confidence interval of −0.20 to 0.19 (*p* = 0.96), and significant heterogeneity among the studies was observed (I^2^ = 63%, *p* = 0.07).

At 6 and 18 h, only one study assessed fibrinogen, showing no better values between groups. Lastly, no statistically significant differences were found between groups (*p*-value = 0.67) (Figure S4).

Regarding D-dimer, it was evaluated in a total of three studies, involving 410 participants (205 in the Cell Salvage group and 205 in the control group). Two studies showed a high risk of bias [39,41], while the third study demonstrated a low risk of bias [54]. In the immediate postoperative period, two studies showed better results for Cell Salvage, and the third did not show a statistically significant association, with the diamond touching the no-effect line. An MD of −0.38 was obtained, with a 95% confidence interval of −0.73 to −0.02 (*p* = 0.04), and there was significant heterogeneity among the studies (I^2^ = 52%, *p* = 0.12). At 6 h, 18 h, and 24 h, only one study assessed D-dimer, showing a trend of better values for the control group. Lastly, no statistically significant differences were found between groups (*p*-value = 0.60) (Figure S5).

Finally, for the INR, the two identified studies included a total of 1096 participants, with 534 in the Cell Salvage group and 562 in the control group, showing a high risk of bias [43,59]. In the immediate postoperative period, both studies showed better INR levels for the control group. An MD of 0.06 was obtained, with a 95% confidence interval of 0.03 to 0.10 (*p* < 0.001), with high heterogeneity among the studies (I^2^ = 75%, *p* = 0.05). At 24 h, the same pattern as in the immediate postoperative period was observed, showing better results in the control group. The MD was 0.04, with a 95% confidence interval of 0.02 to 0.07 (*p* < 0.001), with no heterogeneity among the studies (I^2^ = 0%, *p* = 0.73). At discharge, only one study assessed the INR, obtaining better results for Cell Salvage. Ultimately, no statistically significant differences were observed (*p* < 0.35) (Figure S6).

## 4. Discussion

The results show that Cell Salvage does not present statistically significant differences in relation to blood loss in the postoperative period. In fact, during the first 6 h, 12 h, and 24 h after surgery, they present similar losses. However, in the red series, a greater effectiveness of Cell Salvage was observed in terms of the increase in hemoglobin and hematocrit in the patient, above the normal range. This is significant because, given the dynamics of fluids, with blood being a non-Newtonian fluid, the increase in hematocrit figures allows the viscosity to increase in turn. Taking this increase as a reference, it has a proportional effect on a greater adherence in the circulatory stream, with the negative consequences that derive from it.

Furthermore, the results show that, between the groups (control/experimental), as well as their divergence in analytical values, not only did the hematocrit and hemoglobin increase, but there were significant differences in the PT, D-dimer, total PT, and aPTT values post-surgery. This suggests that, although Cell Salvage does not directly affect hemorrhage, it does modify other blood parameters, especially those affecting coagulation. Similar results were obtained for platelets, FFP, and fibrinogen, where there was also a significant statistical difference between the two groups, although it is true that the results were better for the control group in the INR measurement.

These facts present us with a turning point on the operability of the electronic device, especially knowing when it should be used and what consequences are associated with its use. For this reason, it is necessary to discuss in detail each of the most striking and necessary results for the knowledge of the scientific community.

### 4.1. Cell Salvage-Associated Hemorrhage and Red Series Effectiveness

Bleeding, with its different locations and ranges of severity, is a relatively frequent cause in this type of extracorporeal surgery; however, it should also be noted, from the point of view of the use of the cell saver, that the results obtained did not reveal statistically significant differences, nor was there even a firm and clear tendency to obtain benefits with the use of this device. In contrast, a previous meta-analysis found that people who had undergone cardiac surgery with this device had a 31% decrease in the risk of requiring a CBR blood transfusion [29]. However, the authors report a large heterogeneity in the clinical trials included in their study, as is the case in the present meta-analysis. This situation undermines the possibility to extrapolate the results and to determine the exact effectiveness that the use of Cell Salvage may have in the control or prevention of bleeding.

In contrast, other cohort studies by Vonk et al. found that the mean number of transfusions in control patients was higher when compared to Cell Salvage patients (control: mean 2, 95% CI: 1–5 vs. Cell Salvage: mean 1, 95% CI: 0–3; *p*-value < 0.001). Similarly, they assessed postoperative blood loss. The authors concluded that these losses were lower in the Cell Salvage group compared to control patients at 6, 12, and 24 h postoperatively [64]. These results are in line with Bauer et al. at 6 h and Niranjan et al. at 24 h but do not agree with the other authors included in this review [38,43,45,54,55,57,59,60,63]. These differences may stem from a lack of consensus in the protocols, variations in the perfusionist’s expertise, or differences in the comorbidities of the patients included.

Another associated alteration is the irreversible modification of the structure of the blood components due to their interaction with the biomaterials that make up the shunt circuit, which may alter their function [65]. This does not suggest that technological advances will be able to remedy these alterations; however, if they are not published in the scientific evidence, it will be difficult to contribute to the development of new devices to alleviate this deficit.

### 4.2. Hemoglobin and Hematocrit Levels after Use of Cell Salvage

The American Heart Association (AHA) [66] states that hemoglobin levels of 12 g/dL and hematocrit of 28% are considered adequate in cardiac surgery, with other studies [67] supporting this position. In our meta-analysis, only two studies met the hemoglobin criteria, one being in the immediate postoperative period [49] and the other trial at 24 h [61].

Regarding hematocrit, all the patients in the Cell Salvage group had percentages above 28%. However, if we take into account the criteria where hemoglobin less than 9 g/dL and hematocrit less than 27% are considered strong candidates for transfusion of blood or blood products [68], we would only have one study in the immediate postoperative period in the Cell Salvage group that would require an additional allogeneic transfusion [54] compared to the three studies requiring such transfusion in the control group [41,44,54]. This is in agreement with the results obtained by Vonk et al., (2015), where the Cell Salvage group had a slightly higher hemoglobin level at discharge (6.9 ± 0.7 mmol/L vs. 6.7 ± 0.7 mmol/L; *p* < 0.05) than those in the control group [64]. This is refuted by the more recent trial conducted by Tachias et al. in 2022, where it was observed that the mean postoperative hemoglobin concentrations at 24 h and their postoperative values were in favor of patients in the Cell Salvage group [59]. The authors explain the results by noting the relatively low concentration, with a hematocrit level close to 42%, compared to the hematocrit > 50% indicated in the manufacturer’s specifications. Specifically, the type of device used at the time of the surgery in question (as there are different electronic devices on the market), as well as the operability according to the practitioner using it, produced a hemoglobin concentration effect of 150% [69]. This fact, combined with a shorter lifespan of the recovered red blood cells [47,70], could have contributed reciprocally to the comparable red blood cell transfusions between the different groups.

This observation is not novel, as several studies in the last decade [58,71,72], as well as one of the most recent meta-analyses, have shown that cell recovery did not influence the number of red blood cells transfused. They also suggested a trend, although not statistically significant, towards greater use of fresh frozen plasma (FFP) and platelets [29,72]. In contrast, another meta-analysis from 2018 found that cell salvage reduces both the percentage of patients transfused during the perioperative period and the volume of allogeneic blood products administered [20].

### 4.3. Effectiveness of Cell Salvage on Clotting Times

One of the most controversial points is the coagulation parameters and the use of Cell Salvage, given the variability of results across studies. In 2022, Tachias et al. reported low platelet and fibrinogen concentrations and an undetectable INR > 10 and aPTT (>180 s). A decrease in the platelet count at ICU admission was observed in both groups, in contrast to other techniques as found by Boyle et al. with ultrafiltration using the HemoSep© device [39] or by authors who found an increased platelet content and preserved platelet functionality as assessed by thromboelastography [73].

Similarly, Rubens et al. performed a cardiotomy trial that revealed prolongation of the INR and thrombin time and reductions in fibrinogen levels for at least 12 h postoperatively [74], while other authors described a consumption coagulopathy in the Cell Salvage group [58] and a significant decrease in several coagulation factors (I, II, VII, XI, XIII) in the Cell Salvage concentrate [75].

Therefore, due to activated coagulation combined with accelerated fibrinolysis and postoperative bleeding in their Cell Salvage group, some investigators called for a more cautious use of Cell Salvage techniques in patients at high risk of perioperative bleeding [58], while others indicated higher costs in Cell Salvage [60] and recommended avoiding re-transfusion volumes higher than 1 L [76], despite the recommendation of widespread use by several scientific societies [76,77,78,79,80]. In fact, scientific evidence already exists, such as the study by Luque, who specified the red blood cell reinfusion threshold with the Cell Salvage device based on a specific volume of blood in the reservoir to be processed, which has greater or lesser repercussions for the patient, as well as the relationship between the processing time and obtaining the reinfusion volume [49].

From a critical perspective, the impact of Cell Salvage on coagulation times highlights key clinical challenges. The variability in study outcomes underscores the need for standardized protocols, as some research indicates significant alterations in coagulation parameters, such as a prolonged INR and reduced fibrinogen levels, while others do not. This inconsistency may be influenced by differences in devices and patient characteristics, particularly in those at high risk of perioperative bleeding.

Although Cell Salvage can reduce the need for allogeneic transfusions, the potential for coagulation disturbances poses a risk, especially for vulnerable patients. A cautious, individualized approach is essential, balancing the hematological risks with potential benefits. Technological improvements should focus not only on efficiency but also on minimizing adverse effects on coagulation. Studies such as Luque’s offer a path toward more evidence-based, tailored thresholds for reinfusion, which could enhance clinical outcomes.

On the other hand, our results are in line with the meta-analysis by Al Khabori M et al., where no increase in the rate of platelet transfusion or FFP was found [29]. However, the same authors in a previous cohort study observed an increased risk of FFP transfusion and increased platelet transfusion rate. The latter would be in line with the hypothesis of hemostasis triggered either by profuse bleeding or by extracorporeal reinfusion of large amounts of blood [72]. One of the factors that may influence this lack of consensus in the studies carried out seems to be caused by the process of massive heparinization with subsequent administration of protamine to which cardiac surgery patients are subjected. Both drugs are associated with significant alterations in platelet function and fibrinolysis. It should be noted that, in this type of surgery under an extracorporeal circuit, the pulmonary or minor circulation is no longer active, as the circuit itself acts as an exchanger of the blood–gas barrier. As a result, the inactivity of the lungs during the time of connection to the extracorporeal machine means that the lungs act as a reservoir, allowing part of the heparin to lodge, and once it has left the extracorporeal circuit, and after the administration of protamine (heparin antagonist), the heparin returns to the bloodstream, which may cause alterations in platelet function [81,82].

Another factor to consider is the type of electronic device that each hospital has and, more specifically, the professional who uses it. These devices can operate continuously or discontinuously, with a high margin of variability in terms of the revolutions per minute in the centrifugation chamber, the crystalloid used with its corresponding heparin to prevent clotting of the aspirated blood, the vacuum pressure activated, and even the moment when the recovered and processed red blood cells are reinfused. These differences between the devices are so significant that there are already studies that address them in part, such as the study by Wang et al. (2012), where they evaluated three types of Cell Salvage (Cell Saver 5+, Haemonetics; autolog, Medtronic; and CATS Fresenius HemoCare). The authors concluded that the function of washed red cells and the efficacy in removing waste products differed widely from one device to another (*p*-value = 0.021 for hematocrit and *p*-value = 0.008 for hemoglobin). In our meta-analysis, six different types of brand names were identified, with C.A.T.S., Fresenius Hemocare GmbH©, Bad Homberg, Germany (*n* = 7), and Haemonetics, Braintree, MA, USA (*n* = 5) being the most represented [62]. In addition, each of their own characteristics must be taken into account, which has a direct impact on the quality of the red blood cells obtained.

### 4.4. Gender Differences in Cell Salvage Effectiveness

Despite the heterogeneity of the product, it is important to note that no studies have been identified that address gender differences in the effectiveness of cell salvage, although several studies have found that women are at greater risk of bleeding and transfusion. These notable differences may be due to the disparity in the size of blood vessels, cardiovascular system organs, or anatomical disposition, as their smaller size (given their femininity) has a direct impact on the choice of tubing for the extracorporeal circuit and thus directly affects fluid dynamics. It must be taken into account that the larger the diameter of the tubing chosen to connect the patient to the extracorporeal circuit is, the higher the fluid pressure, the higher the viscosity of the blood, the lower the adherence, and the lower the velocity. In addition, the smaller the diameter is, the lower the pressure and the greater the resistance, speed, and adherence. All this makes it necessary to calculate the theoretical priming volume of the machine, as well as the hemodilutional volume. For this reason, the patient’s cardiac output and body surface area index are always taken into account.

To this must be added the fact that women have a different blood volume due to their different body size, as well as the losses that occur monthly with menstruation, which leads to unequal hemoglobin levels, increasing the risk of anemia in women. These aspects have not been taken into consideration in any of the studies included, so we believe that they should have analyzed the results separately, setting specific reference values for women and men. In addition, there are other factors such as hormonal fluctuations in the different stages of the menstrual cycle or other reproductive stages such as pregnancy or menopause that have also not been considered [83,84,85,86].

### 4.5. Limitations and Strengths

The results of this meta-analysis should be interpreted with some caution. The sample size of most of the studies included in the analyses was small. The mean number of patients was 120 subjects ± 84 (95% CI 145 to 76; Max: 352 Min: 20). Studies have shown that the results of meta-analyses involving trials with small numbers of patients may lead to confounding bias compared to trials with a large sample size [87,88].

In addition, the heterogeneity found in some of the analyses makes it difficult to interpret the results. There was no obvious cause for the heterogeneity, but it is possible that it reflects the lack of working protocols on the use of cellular rescue, especially with regard to the type of device chosen and how it is used. In addition, because it is difficult to blind the intervention, practitioners’ behavior could have been driven by knowledge of group allocation, especially when transfusion guidelines were not established. The decrease in the proportion of patients receiving allogeneic transfusion may be related to the intrinsic efficacy of Cell Salvage or to clinician transfusion behavior, especially when transfusion guidelines have not been established. Because most studies do not state their transfusion protocols, nor do they indicate how rigorously these protocols were followed, it is possible that there was a difference in the indications for the use of Cell Salvage, which could have favored the Cell Salvage group.

Another very important limitation concerns the use of the Cell Salvage. As the authors do not state how the device is used (continuous or discontinuous), their results have serious interpretation biases. The variability in the way the cell saver is used leads to different results. There are already studies that deal with this, as well as the different retriever devices, which have different characteristics and thus bias the results of the studies. Therefore, it is crucial to develop and adhere to standardized protocols for the use of Cell Salvage and transfusion practices. This includes clear guidelines on when and how to apply Cell Salvage techniques, which can minimize variability in the results due to different practices among clinicians.

Regarding recommendations for future cellular retriever research, we recommend that implementing strategies to blind intervention assignments whenever possible could help mitigate bias introduced by the practitioners’ knowledge of group assignments. Improved randomization techniques could also enhance the validity of the results. In addition, future studies should require the inclusion of detailed transfusion protocols to facilitate the comparison and synthesis of results across studies. This would help identify whether variations in protocols contribute to differences in outcomes.

Similarly, conducting longitudinal studies could provide information on the long-term effects of Cell Salvage and transfusion practices, thereby enriching the understanding of their efficacy over time. Therefore, it is recommended that patients be monitored when they are transferred to the hospital ward or post-surgical recovery unit.

On the other hand, including diverse patient populations in studies may help generalize the findings and assess the effects of biological sex and other demographic factors more accurately.

The strengths of this meta-analysis include the clear inclusion and exclusion criteria, a comprehensive literature search, and large subgroup analyses. In addition, all studies identified in languages other than English (German, French, Spanish, Italian, Danish, and Norwegian) were included because the exclusion of articles for linguistic reasons was found to affect the results of meta-analyses [89,90]. In addition, the biological sex perspective was assessed according to the new international recommendations, and a large gap was observed between men and women. Furthermore, above all, this research contributes to a growing body of knowledge with a disparity of results, which makes this meta-analysis even more important for the scientific community.

### 4.6. Clinical Prospective

The need to unify protocols to determine the effectiveness of Cell Salvage to improve biochemical patterns is evident, especially to elucidate those points where there is great controversy such as platelet consumption and coagulation times. These devices present different options for use (continuous/discontinuous; with higher/lower processing revolution; with higher/lower volume of crystalloid mixing, etc.) given their broad portfolio of operational characteristics. Therefore, the volume of blood processed and the volume obtained from the flow recuperator depend on the perfusionist’s knowledge and experience. From this, their performance is derived, and thus, the re-infusion of high-quality red blood cells in turn allows for a higher consumption of the patient’s reserve of platelets and clotting factors. This leads to significant differences in analytical values and, hence, the possible consumption of clotting factors, plasma, or platelets. The use of blood spilled during surgery or from CPB machines can activate hemostatic pathways and lead to low-grade disseminated intravascular coagulopathy in patients receiving this blood, especially if it is received in large volumes [29].

It would be interesting to have the option of manufacturing a device that obtains other components such as platelets, plasma, or coagulation factors from the patient themself, thus saving indirect and direct bench costs. On the other hand, most studies do not indicate the type of priming machine used by perfusionists, which directly influences the hemodynamics of the patient and indirectly influences the use of Cell Salvage in terms of hemoglobin and hematocrit figures.

On the other hand, it would be of interest for studies to homogeneously determine erythrocytes, strain rate, hematocrit viscosity, 2,3 diphosphoglycerate, hematocrit, hemoglobin, free hemoglobin clearance, glucose, lactate, and urea nitrogen, among others. In addition, international measures should be used to express the results. Similarly, patient comorbidities should be identified in order to make more accurate comparisons. Additionally, a protocol for common use by perfusionists should be established, which includes the choice of Cell Salvage.

## 5. Conclusions

The use of the Cell Salvage system has great uncertainty in the improvement of biochemical parameters, primarily clotting times. The results do not provide definitive evidence regarding its efficacy in the control of bleeding, platelet count, fresh frozen plasma, and fibrinogen. Therefore, it is recommended to increase the number of studies to assess the impact of the Cell Salvage system on improvements in patients’ red blood cell counts and coagulation patterns. Furthermore, protocols should be standardized, and variables such as the sex of the participants, type of device used, and time of reinfusion of the red blood cells obtained should be taken into account, as well as standardizing the protocols of action by those in charge of these devices, in particular perfusionists. For all these reasons, we can affirm that caution should be exercised today in its indiscriminate use until its benefits (with all the aforementioned items) with respect to allogeneic transfusion can be confirmed.

## Figures and Tables

**Table 1:** Characteristics of the included studies.

Study (Author/Year) Evidence Level	Country	Design	Intervention	Sample (M/F) Average Age	Objectives	Implementation Details	Outcomes	Parameters Assessed
Bauer et al. (2017) (1+) [38]	Germany	Prospective, randomized, controlled clinical trial	CS (MiECCB System©) No CS (control)	30 (23/7) 36 (29/7) CS: 67.5 ± 9.9 No CS: 66.7 ± 8.8	To investigate the impact of cell washing shed blood from the operating field versus direct return to the ECC on the biomarkers for systemic inflammation.	CS: Suction blood was separated, and CS was performed before the blood was re-transfused as an autologous RBC concentrate. Control: The suction blood was separated and directly re-transfused without any treatment.	CS has positive effects on hemorrhage.	Hemorrhage HB
Boyle et al., (2019) (1+) [39]	United Kingdom	Randomized clinical trial	CS (HemoSep©) No CS (control)	26 (NR) 28 (NR) Age (NR)	To assess homeostatic markers in patients who underwent surgeries where they received conventional CS.	CS: Patients allocated into CS group, blood from the bypass reservoir was drained into two treatment bags with half the volume in each. Control: Received autologous blood.	CS device demonstrated a slight increase in hemostatic markers in low-risk cardiac surgery.	aPTT PT Fibrinogen D-dimer
Campbell et al., (2011) (1++) [40]	United Kingdom	Randomized clinical trial	CS (C.A.T.S., Fresenius Hemocare GmbH©, Bad Homberg, Germany) No CS (control)	10 (9/1) 10 (9/1) CS: 62.0 ± 10 No CS: 64.0 ± 10	To examine the relationship between processing the residual CPB volume and the viscoelastic properties of clot formation in patients undergoing cardiac surgery.	CS: In this group, a continuous autotransfusion system was used prior to the administration of heparin and after reversal with protamine, and the residual CPB volume was processed before transfusion after bypass. Control: CS was not used in this group, and the residual CPB volume was transfused unprocessed after protamine administration.	There was a strong association between clot formation time after surgery and blood loss. The increase in blood loss was 4.1 mL for every one-second increase in clot formation time. CS of the residual cardiopulmonary bypass volume reduced platelet numbers and prolonged clot formation time and maximum clot firmness was less in this group.	HB Platelet count
Daane et al., (2003) (1−) [41]	The Netherlands	Prospective randomized clinical trial	CS (Haemolite 2plus, Haemonetics Corp., Braintree, MA, USA©) No CS (control)	20 (11/9) 20 (13/7) CS: 65.0 ± 10 No CS: 65.0 ± 14	To compare the effects of the transfusion of unprocessed and CS volume on hemostasis, complement activation, postoperative blood loss, and transfusion requirements after elective cardiac surgery.	CS: Patients in this group were transfused with processed blood using a CS device and the residual CPB volume. Control: Patients were transfused with unprocessed residual volume obtained from the extracorporeal circuit.	Processing CPB volume in combination with processing preoperative blood loss may result in reducing the volume of transfusion needed of allogeneic blood product.	HB HCT aPTT PT TT Platelet count Fibrinogen D-dimer
Damgaard et al. (2006) (1++) [42]	Denmark	Randomized clinical trial	CS (Autolog Medtronic, Minneapolis, MN, USA©) No CS (control)	30 (19/11) 30 (16/14) CS: 77.0 ± 3.70 No CS: 76.0 ± 6.70	To clarify the effect of using a CS intraoperatively.	CS: Patients in this group were transfused with blood processed with a blood CS, device residual CPB volume. Control: Patients were transfused with unprocessed residual volume obtained from the ECC.	Use of CS reduced intraoperative RBC loss and seemed to reduce transfusions by 1 unit per patient.	HB HCT
Djaiani et al., (2007) (1−) [43]	Canada	Randomized clinical trial	CS (Fresenius corporation, Concord, CA, USA©) No CS (control)	112 (100/12) 114 (103/9) CS: 67.5 ± 6.0 No CS: 67.0 ± 6.1	To determine whether the replacement of cardiotomy suction with a continuous-flow CS device would improve neuroprotection by minimizing cerebral microembolization and reduce cognitive decline in elderly patients after coronary artery bypass graft surgery.	CS: The continuous-flow CS was used to process shed blood before returning it back to the patient. Control: Cardiotomy suction was used in a standard closed venous reservoir where cardiotomy blood was collected and reinfused through the arterial circuit back to the patient.	Processing of shed blood with CS results in a clinically significant reduction in postoperative cognitive dysfunction after cardiac surgery.	Hemorrhage HB aPTT FFP Platelet count INR
Engels et al. (2016) (1++) [44]	The Netherlands	Randomized, prospective, multicenter clinical trial	CS (Continuous AutoTransfusion System Fresenius©) No CS (control)	99 (69/30) 96 (62/34) CS: 66.0 ± 10.0 No CS: 68.0 ± 9.0	To assess whether intraoperative CS may reduce lung injury following cardiac surgery by removing cytokines, neutrophilic proteases, and lipids that are present in cardiotomy suction blood.	CS: Blood was collected from skin incision until closure of the sternum including cardiotomy suction blood and residual heart–lung machine blood processed with a CS device. Control: Conventional cardiotomy suction device was used, and the residual blood from the heart–lung machine was re-transfused to the patient through a standard blood transfusion set.	First, patients in the cell saver group had higher HB levels during the first 24 h after surgery; however, the perioperative packed RBC transfusion rates were similar between the 2 groups. Second, patients in the CS group had a higher INR ratio and lower platelet count at the time of ICU admission.	HB Platelet count
Goel et al., (2007) (1+) [45]	India	Prospective randomized trial	CS (Dideco, Mirandola, Italy) No CS (control)	24 (21/3) 24 (21/3) CS: 58.2 ± 8.7 No CS: 61.9 ± 10.0	To evaluate the safety and efficacy of this modality in patients undergoing off-pump coronary artery bypass grafting.	CS: CS was used to salvage and autotransfuse shed blood from the time of incision. Control: This group was administered banked homologous packed RBCs as the only blood replacement therapy and served as the control.	The use of CS reduced the requirement for HB transfusion. Its use is not associated with any clinically significant bleeding diathesis.	HB FFP
Hogan et al. (2015) (1−) [46]	United Kingdom	Randomized controlled trial	CS (HemoSep©) No CS (control)	25 (19/6) 28 (24/4) CS: 70.5 ± 10.2 No CS: 67.7 ± 10.2	To compare the autotransfusion of residual CPB blood with residual blood concentrated using the novel HemoSep© device.	CS: In patients allocated to the CS group, blood from the bypass reservoir was drained into two treatment bags with half the volume in each. Control: The blood was re-transfused to the patient at a rate determined by the anesthetist.	There was no difference in the HB concentration in both groups. HemoSep© reduced the weight of the blood in comparison to the control group.	HB aPTT PT Platelet count
Klein et al., (2008) (1++) [47]	United Kingdom	Randomized clinical trial	CS (C.A.T.S—Fresenius Hemocare, France) No CS (control)	111 (84/27) 102 (78/24) CS: 67.4 ± 10.2 No CS: 68.6 ± 9.6	To examine the hypothesis that the use of CS during and after routine cardiac surgery would reduce the proportion of patients exposed to allogeneic blood transfusion.	CS: The CS was prepared before the start of surgery, and it was used exclusively for surgical suction before and after CPB in the CS group. Control: After CPB, any remaining blood in the bypass machine tubing and reservoir was collected in a bag and transfused directly to the patient.	There was no difference between the two groups in the proportion of patients exposed to allogeneic blood (32% in both groups, relative risk 1.0 *p* = 0.89).	FFP
Laub et al. (1993) (1−) [48]	USA	Randomized clinical trial	CS (Cell Salver 4, Haemonetics Corp.) No CS (control)	19 (15/4) 19 (15/4) CS: 65.0 ± 2.4 No CS: 64.4 ± 2.1	Assessing intraoperative autotransfusion	CS: The shed intraoperative and pump blood remaining at the conclusion of bypass was washed with CS and autotransfused. Control: Shed blood was discarded, and pump blood was re-transfused without washing.	Intraoperative use of CS decreased the volume of blood products required.	FFP
Luque et al. (2018) (1−) [49]	Spain	Randomized clinical trial	CS (Continuous Autologous Autotransfusion System, C.A.T.S, Fresenius) No CS (control)	80 (63/17) 91 (78/13) CS: 65.0 ± 6.2 No CS: 67.0 ± 5.4	To analyze the evidence on the effectiveness of CS to reduce blood transfusions.	CS: Lost blood was recovered from the operative field with CS, and after processing, the obtained RBCs were reinfused into the patient continuously during surgery. Control: Traditional transfusion system.	Both HB and HCT were lower in the control group in comparison to the CS group, both during and after surgery. Blood transfusions were higher in the control group both during and after surgery.	HB HCT Platelet count
Marchiex et al. (2008) (1+) [50]	Canada	Randomized clinical trial	Groups A and B: Non-CS. Groups C and D: CS.	A: 25 (22/3) B: 25 (24/1) C: 25 (21/4) D: 25 (22/3) A: 60 ± 6.1 B: 61.2 ± 7.1 C: 59.7 ± 8.5 D: 59.2 ± 9.7	To determine the effect of processing of pericardial blood with a CS and vacuum-assisted cardiopulmonary bypass (VACPB) on the reduction of postoperative inflammation.	Group A: No CS and no VACPB. Group B: VACPB alone. Group C: CS alone. Group D: CS and VACPB. The processed blood in groups C and D was re-transfused immediately after weaning from CPB. Patients in groups A and B received re-transfusion of suctioned blood during CPB.	The use of CS decreases the activation of the complement alternative pathway and reduces neurologic injury and exposure to allogeneic blood transfusion.	FFP
McGill et al. (2022) (1−) [51]	USA	Randomized clinical trial	1–Intraoperative CS (Dideco Compact, Dideco, Mirandola, Italy), intraoperative 2–CS with acute perioperative normovolemic hemodilution, 3–No CS (control)	84 (75/9) 84 (74/10) CS: 63.8 ± 7.8 No CS: 63.4 ± 9.1	To assess the effectiveness of two mechanical methods of blood conservation in reducing the need for allogeneic RBC or coagulation products during cardiac surgery.	CS: Intraoperative blood salvage with CS. This blood was re-transfused at the termination of bypass. Control: Shed blood was discarded.	The need for allogeneic RBC transfusion in elective coronary artery bypass grafting can be reduced by using intraoperative CS.	FFP
McShane et al., (1987) (1−) [52]	Ireland	Clinical trial	CS (Continuous Autologous Autotransfusion System, C.A.T.S, Fresenius) No CS (control)	20 (12/8) 21 (16/5) Age (NR)	To measure HB, HCT, pH, and the concentrations of lysozyme, potassium, and red cell 2,3-DPG, and to compare these values with those in donor blood.	CS: An intra-surgical CS was used. Control: A conventional pressure suction drain was used.	The autotransfusor is a useful aid for blood conservation, producing good quality RBCs with relatively normal pH and potassium values. However, modification of the centrifugation and washing is required to lessen the high white cell count and heparin concentrations found in the saved blood.	HB HCT
Merville et al., (1991) (1−) [53]	France	Prospective randomized clinical trial	CS (Haemonetics^®^) Control: filter	60 (44/16) 60 (41/19) CS: 61.0 ± 11.0 No CS: 63.4 ± 10.0	To determine, in adult cardiac surgery, the effectiveness of two intraoperative blood collection techniques.	CS: The residual blood of the circuit of the circulatory system is also treated by CS. This treated blood can be returned to the patient in the operating room or during their transfer to the ICU. Control: The residual blood of the extracorporeal circuit is ultrafiltered. The blood reconcentrated by gentle hemofiltration is collected in a transfer bag. This blood is returned to the patient in the ICU.	The use of CS associated with the use of normovolemic hemodilution was more effective. It makes it possible to carry out 65% of cardiac surgery interventions under extracorporeal circulation without homologous blood supply.	FFP
Murphy et al., (2004) (1+) [54]	United Kingdom	Randomized controlled trial	CS (Autolog Medtronic, Watford, UK) No CS (control)	97 (74/23) 99 (86/13) CS: 62.3 ± 18.7 No CS: 64.3 ± 9.2	To compare the effects of autotransfusion of washed salvaged RBCs on coagulation pathway function and blood loss after cardiac surgery in a randomized controlled trial.	CS: All blood loss from skin incision to commencement of CPB and then after administration of protamine to skin closure was salvaged at high pressure suction. All blood remaining in the CPB circuit after discontinuation of bypass was re-transfused. Control: All blood spilled before commencement of CPB and after administration of protamine was aspirated using a high-pressure sucker and discarded.	Autotransfusion is a safe and effective method of reducing the use of homologous bank blood after routine first-time coronary artery bypass grafting.	Hemorrhage HB aPTT ratio Platelet count D-dimer
Murphy et al., (2005) (1+) [55]	United Kingdom	Randomized controlled trial	CS (Dideco, Gloucester, UK) No CS (control)	30 (25/5) 31 (23/8) CS: 66.4 ± 7.6 No CS: 62.3 ± 9.3	To evaluate the safety and effectiveness of intraoperative CS and autotransfusion of washed salvaged RBCs after first-time coronary artery bypass grafting performed on the beating heart.	CS: Patients underwent intraoperative CS, with autotransfusion of washed, salvaged RBCs at the completion of the operative procedure. All blood lost, from skin incision to skin closure, was salvaged at high-pressure suction, washed, and autotransfused. Control: All blood spilled, from skin incision to skin closure, was aspirated with a high-pressure sucker and discarded.	The postoperative HB concentration was significantly higher in the autotransfusion patients. HB levels were similar in the 2 groups after protamine administration and at 1 h (*p* = 0.71 and *p* = 0.60, respectively), but at 24 h, the mean level was, on average, 1.02 g/dL lower in the control group (*p* = 0.0007). A similar difference was noted with HCT. At 24 h, the mean HCT level was significantly lower (0.03 L/L) in the control group (*p* = 0.0008).	Hemorrhage HB HCT aPTT ratio FFP Platelet count Fibrinogen
Niranjan et al., (2006) (1+) [56]	United Kingdom	Randomized clinical trial	CS (Dideco, Gloucester, UK)	20 (16/4) 20 (16/4) CS: 66.3 ± 7.3 No CS: 66.1 ± 10.8	To investigate the potential additive effects of autologous CS blood transfusion and CPB on blood loss, homologous blood transfusion requirements and clotting parameters in patients undergoing CABG for the first time.	CS: The device was used to collect blood lost from skin incision to skin closure in the off-CPB group and from skin incision to commencement of CPB and returned to the venous reservoir. Any remaining blood in the CPB circuit after discontinuation from bypass was re-transfused via the aortic cannula before decannulation. Control: In the off-pump group without CS, all lost blood from skin incision to closure was suctioned with a high-pressure sucker into a waste container. All blood lost from skin incision to commencement of CPB and protamine reversal to skin closure was aspirated into a waste sucker.	Off-pump CABG is associated with a significant reduction in intraoperative mediastinal blood loss and homologous transfusion requirements. Autologous transfusion of salvaged washed mediastinal blood reduced homologous transfusion significantly in the on-CPB group. CS caused no significant adverse impact on coagulation parameters in on- or off-CPB CABG. Postoperative morbidity and blood loss were not affected using CPB or autologous blood transfusion.	Hemorrhage
Reyes et al., (2010) (1−) [57]	Spain	Randomized clinical trial	CS (CATS, Fresenius Hemocare, France) No CS (control)	34 (24/10) 29 (18/11) CS: 65.5 ± 12.1 No CS: 63.7 ± 12.7	To analyze if the use of CS systems reduces the need for blood products in low-risk patients undergoing cardiac surgery.	CS: Device was used in the CS group. CS was used all through the procedure. At the end of surgery, all remaining blood inside the circuits was recovered and concentrated by the CS. All recovered blood was transfused to the patients, and cardiotomy suction was used and the blood transfused to the patient. Control: All blood in the surgical field was aspirated only using the cardiotomy suction.	In low-risk patients, the CS system did not reduce the need for blood transfusion. Clinical outcomes were similar regardless of the use of a CS saver system. A low preoperative HB level and a low BSA were related to the use of blood products.	Hemorrhage HB FFP Platelet count
Scrascia et al. (2012) (1−) [58]	Italy	Prospective, randomized, controlled trial	CS (Hemonetics©) No CS (control)	17 (8/9) 17 (13/4) CS: 71.0 ± 8.0 No CS: 66.0 ± 10	To evaluate the influence of residual pump blood salvage on inflammatory, coagulative, and fibrinolytic system activation and on postoperative HB levels and transfusion rates in patients undergoing coronary artery bypass grafting.	CS: The CS system was used to collect residual blood remaining inside the bypass CPB at the end of the surgery. This blood was transferred into a sterile collecting bag and transfused to the patient via a standard blood-giving set at the time of skin closure. Control: Blood samples were collected from a peripheral arterial line after the induction of anesthesia and 24 h later. Samples were also taken from the collecting bag after the washing and concentration procedure and prior to infusion into the patient.	The recovery of blood with the use of the CS improves postoperative HB levels but induces the generation of thrombin and activation of fibrinolysis, which increase the potential for coagulopathies.	HB FFP
Tachias et al., (2022) (1−) [59]	Greece	Prospective randomized clinical trial	CS (Haemonetics Cell Saver^®^) No CS (control)	99 (75/24) 110 (87/23) CS: 66.3 ± 10.0 No CS: 67.1 ± 10.0	To investigate the potential effects of the centrifuged end-product on bleeding, transfusion rates, and other transfusion-related variables in adult cardiac surgery patients submitted to ECC.	CS: The device was used for all patients and collected lost blood from the moment of pericardiotomy to the ECC, and after ECC weaning to the end of the surgery. The CS concentrate was transfused to the patients. Control: Patients underwent surgery without CS use.	Within the study’s constraints, the perioperative use of the CS concentrate does not seem to affect bleeding or transfusion variables, although it could probably ameliorate postoperative oxygenation in adult cardiac surgery patients. A tendency to promote coagulation disturbances was detected.	Hemorrhage HB aPTT Platelet count Fibrinogen INR
Vermeijden et al. (2015) (1−) [60]	The Netherlands	Multicenter, factorial randomized, partially blinded clinical trial	CS (Continuous Autologous Autotransfusion System, C.A.T.S, Fresenius; Haemonetics, Braintree; Sorin, Milan, Italy). No CS (control)	175 (140/35) 177 (66/111) CS: 66.0 ± 9.5 No CS: 65.0 ± 9.7	To investigate the effect of CS, LD filters, and their combination on transfusion requirements in cardiac surgical patients.	CS group: Cardiotomy suction blood, blood from the surgical field, and residual heart–lung machine blood was collected. This blood was washed in the CS. Control: Neither CS nor a filter was used. A conventional cardiotomy suction device was used, and blood from the surgical field was discarded after the reversal of heparin.	There was no significant effect of CS or the filter on the total number of blood products. Using a CS reduced RBC transfusions within 24 h but not during the hospital stay. The use of a CS was also significantly associated with increased transfusions of FFP and the percentage of patients who received any transfusion but not with platelets, whereas filters were not associated significantly.	Hemorrhage HB
Wang et al., (1994) (1−) [61]	Taiwan	Prospective clinical trial	CS (Haemonetics, Braintree, MA, USA) No CS (control)	41 (35/6) 70 (54/16) CS: 57.8 ± 9.1 No CS: 57.7 ± 7.6	To assess the efficacy of this newly introduced blood conservation technique in terms of reducing postoperative transfusion requirements in two different categories of patients who underwent corrective cardiac surgical procedures.	CS: CS was used as the blood conservation method during surgery. Control: Patients underwent surgery without CS use.	The use of CS did not increase the postoperative chest tube drainage in either the CABG or the redo patients. CS is useful in CABG patients, as far as the reduction in transfusion requirements is concerned.	Hemorrhage HB HCT Platelet count
Wang et al., (2012) (1−) [62]	China	Randomized clinical trial	CS: (Cell Saver 5+; Haemonetics) CS: (autolog; Medtronic) CS: CATS; Fresenius HemoCare)	10 (NR) 10 (NR) 10 (NR) Age (NR)	To evaluate three commercially available CS devices in terms of erythrocyte function and the quality of washed RBCs during CPB.	The salvaged blood was processed by different CS devices, which were set up and operated according to the manufacturers’ recommended instructions. Filling and emptying of the centrifugal chambers were performed in automatic mode.	CS devices use the same theory of centrifugation; however, based on different designs, the function of the washed RBCs and the undesirable content removal efficiency differ widely from one device to another.	HB HCT
Xie et al. (2015) (1−) [63]	China	Prospective, randomized, controlled trial	CS (Haemonetics, USA) No CS (Control)	72 (35/37) 69 (29/40) CS: 51.7 ± 15.6 No CS: 53.1 ± 15.1	To evaluate the efficacy, safety, and cost-effectiveness of intraoperative CS in CPB surgery.	CS: Shed blood from the wound and mediastina were sucked into the CS reservoir. At the end of the surgery, residual blood in the CPB circuit was sucked into the reservoir directly. After being filtrated, centrifugated, washed, and concentrated, the recovered blood became autologous blood and was then transfused back to the patients. Control: Shed blood from the wound and mediastina during the period of non-heparinization and residual blood were sucked into the suction apparatus and were discarded.	The proportion and quantity of perioperative allogeneic RBC transfusion were significantly lower in the CS group. The incidence of residual heparin and total impairment of blood coagulative function in the 24 h after surgery and the incidence of postoperative excessive bleeding were significantly higher in the CS group. The costs of allogeneic RBC transfusion and total allogeneic blood transfusion were also significantly lower in the CS group, but the cost of total blood transfusion was significantly higher in this group.	Hemorrhage FFP

aPTT: Activated Partial Thromboplastin Time; BSA: Body Surface Area; CPB: Cardiopulmonary Bypass; CS: Cell Saver; ECC: Extracorporeal Circulation; ETP: Endogenous Thrombin Potential; FFP: Fresh Frozen Plasma; LD: Leukocyte Depletion; HB: Hemoglobin; HCT: Hematocrit; IAT: Intraoperative Autotransfusion; MiECC System©: Minimal invasive Extracorporeal Technologies; INR: International Normalized Ratio; PT: Prothrombin time; RBC: Red Blood Cell; TT: Thrombin Time. NR: Not Reported.

## Data Availability

Data available on requirement.

## Supplementary Materials

The following supporting information can be downloaded at: https://www.mdpi.com/article/10.3390/jcm13206073/s1, Table S1: Grades of recommendations; Figure S1: Risk of bias graph: review authors’ judgements about each risk of bias item presented as percentages across all included studies. Red = high risk; Green = low risk; Yellow/? = unclear risk; +/− = risk percentage. Figure S2: Risk of bias summary: review authors’ judgements about each risk of bias item for each included study. Red = high risk; Green = low risk; Yellow/? = unclear risk; +/− = risk percentage. Figure S3: Analysis of publication bias by means of a funnel plot; Figure S4: Forest plot presenting fibrinogen over time; Figure S5: Forest plot presenting D-dimer over time; Figure S6: Forest plot presenting INR over time.
