# Supplementary material for: Evaluation of Red Blood Cell Biochemical Markers and Coagulation Profiles Following Cell Salvage in Cardiac Surgery: A Systematic Review and Meta-Analysis
Source: J Clin Med. 2024 Oct 11;13(20):6073. doi: 10.3390/jcm13206073 (PMC11508477; doi:10.3390/jcm13206073)
Supplement: Supplementary file 1 [file jcm-13-06073-s001.zip › Figure S1 Risk of bias graph.pdf]

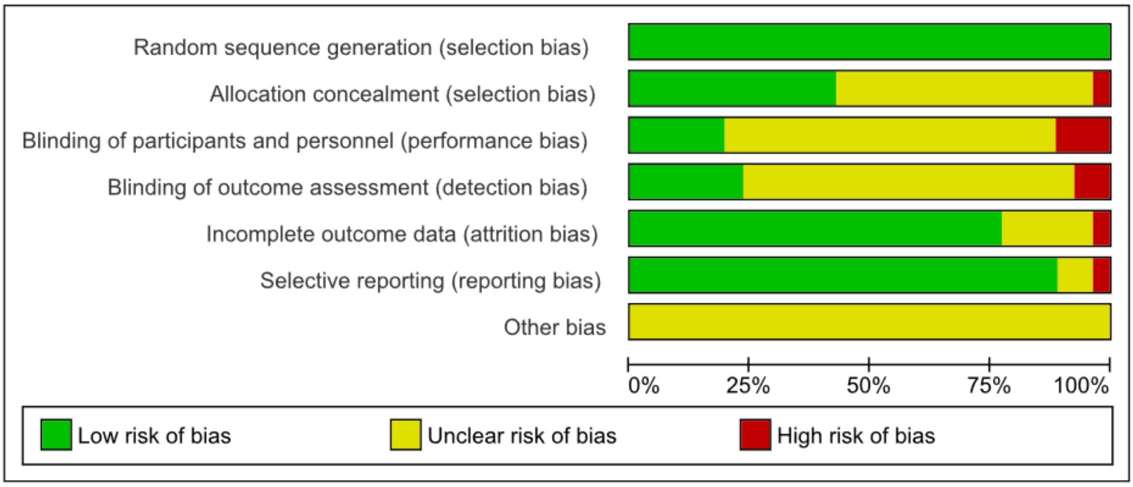

Figure S1. Risk of bias graph: review authors' judgements about each risk of bias item presented as percentages across all included studies. Red=high risk; Green=low risk; Yellow/?=unclear risk; +/−=risk percentage.
