# Supplementary material for: Evaluation of Red Blood Cell Biochemical Markers and Coagulation Profiles Following Cell Salvage in Cardiac Surgery: A Systematic Review and Meta-Analysis
Source: J Clin Med. 2024 Oct 11;13(20):6073. doi: 10.3390/jcm13206073 (PMC11508477; doi:10.3390/jcm13206073)
Supplement: Supplementary file 1 [file jcm-13-06073-s001.zip › Figure S3_ funnel plot.pdf]

Figure S3. Analysis of publication bias by means of a funnel plot

A

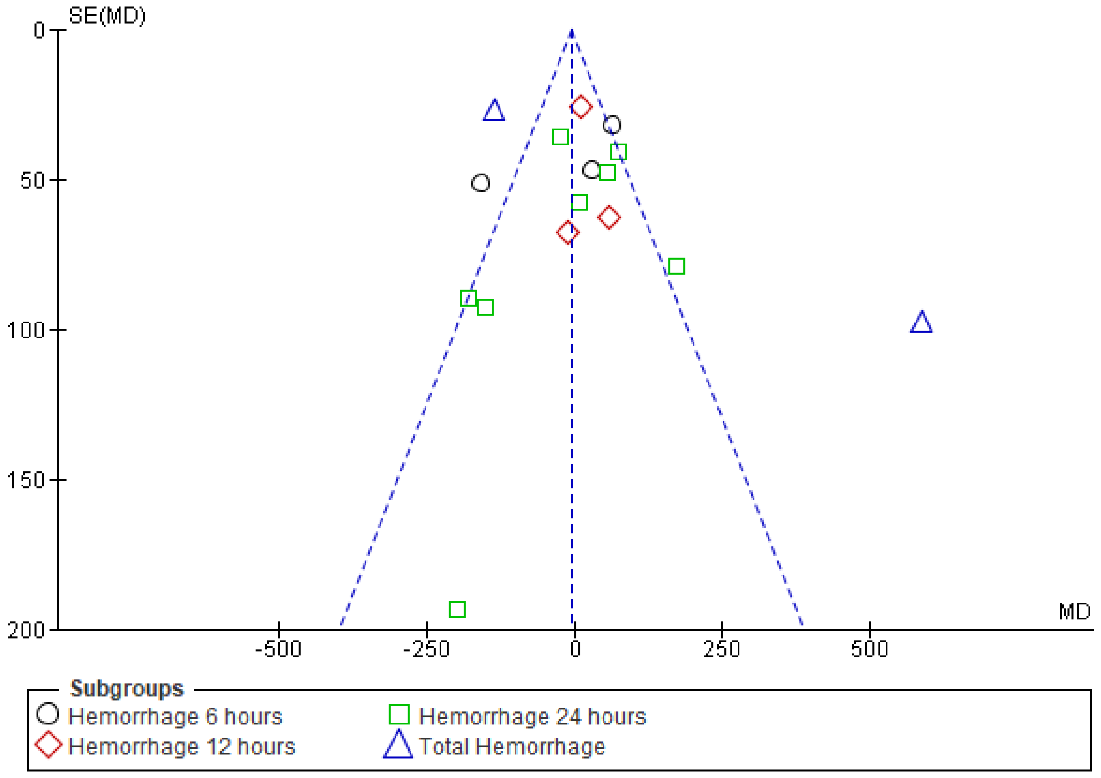


B

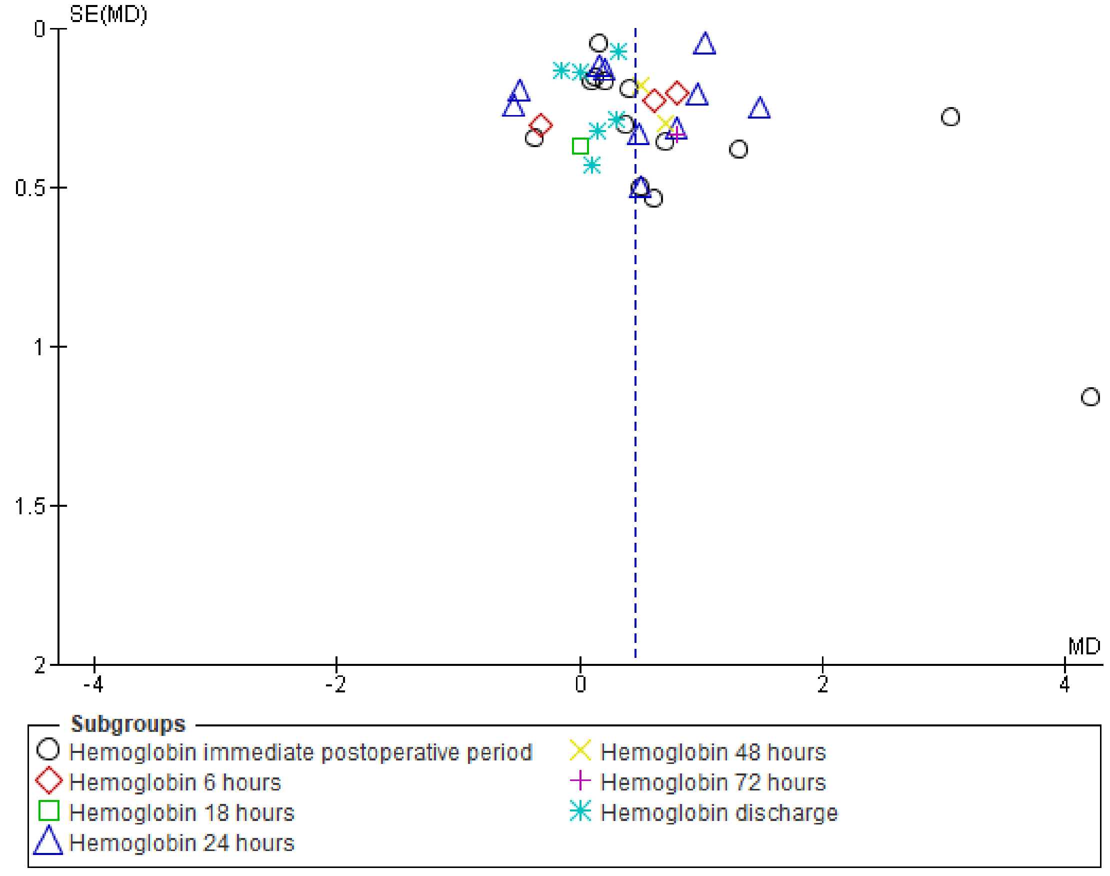

C

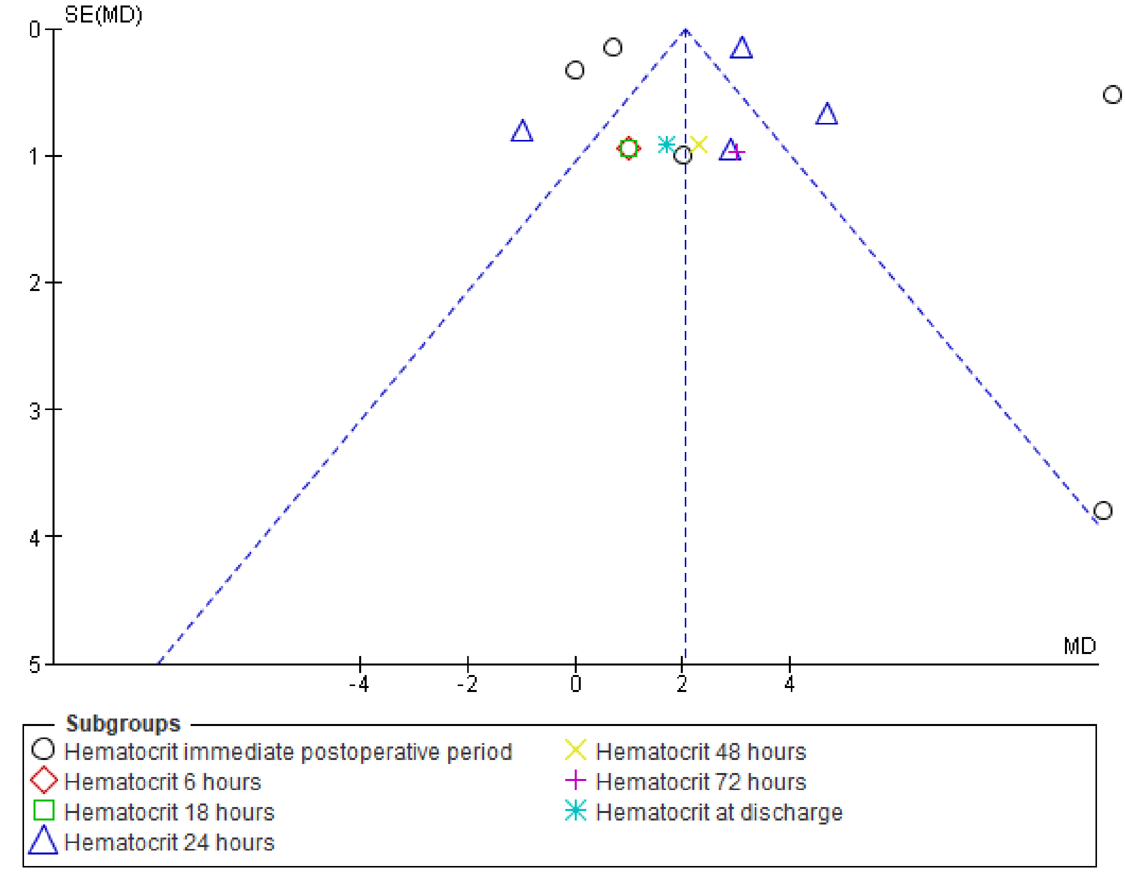
SE(MD)
0
1
2
3
4
5
MD
-4
-2
0
2
4
Subgroups
Hematocrit immediate postoperative period
Hematocrit 6 hours
Hematocrit 18 hours
Hematocrit 24 hours
Hematocrit 48 hours
Hematocrit 72 hours
Hematocrit at discharge

D

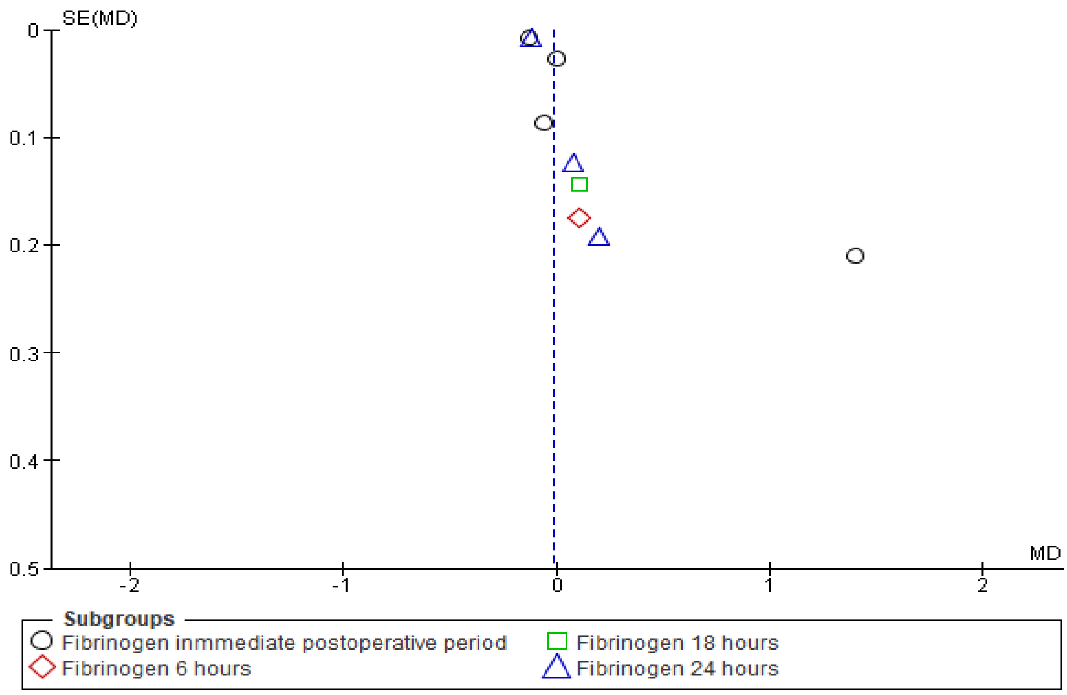
SE(MD)
0
0.1
0.2
0.3
0.4
0.5
MD
-2
-1
0
1
2
Subgroups
Fibrinogen inmmediate postoperative period
Fibrinogen 6 hours
Fibrinogen 18 hours
Fibrinogen 24 hours

E

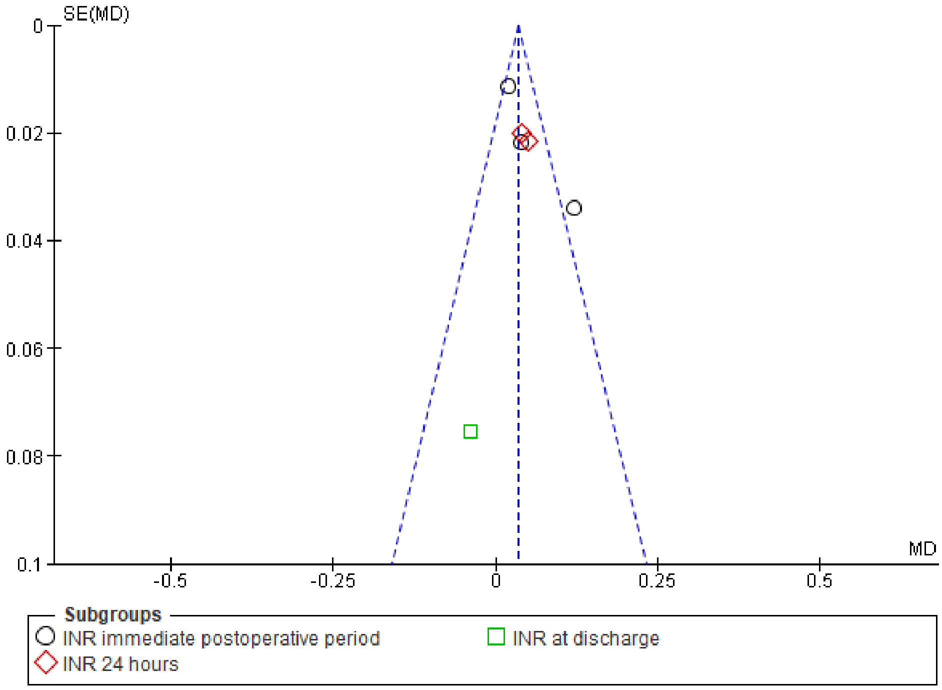
SE(MD)
0
0.02
0.04
0.06
0.08
0.1
MD
-0.5
-0.25
0
0.25
0.5
Subgroups
INR immediate postoperative period
INR 24 hours
INR at discharge

F

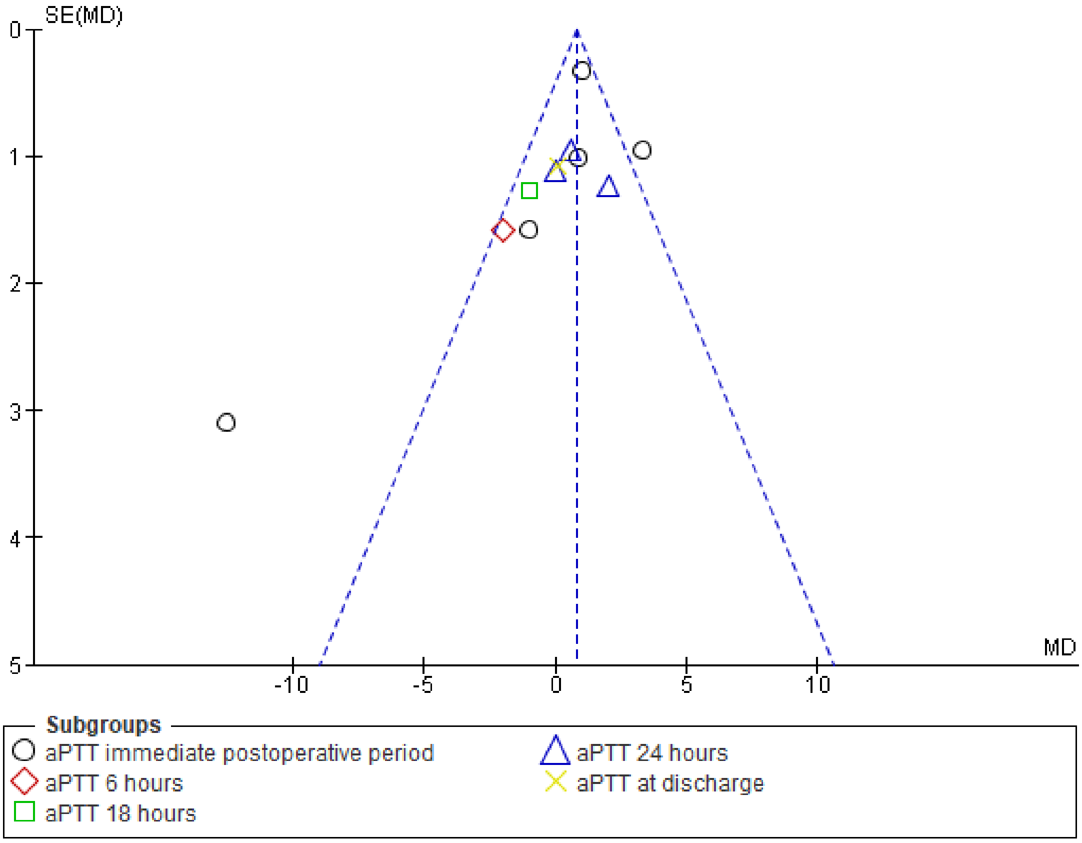
SE(MD)
0
1
2
3
4
5
MD
-10
-5
0
5
10
Subgroups
aPTT immediate postoperative period
aPTT 6 hours
aPTT 18 hours
aPTT 24 hours
aPTT at discharge

G

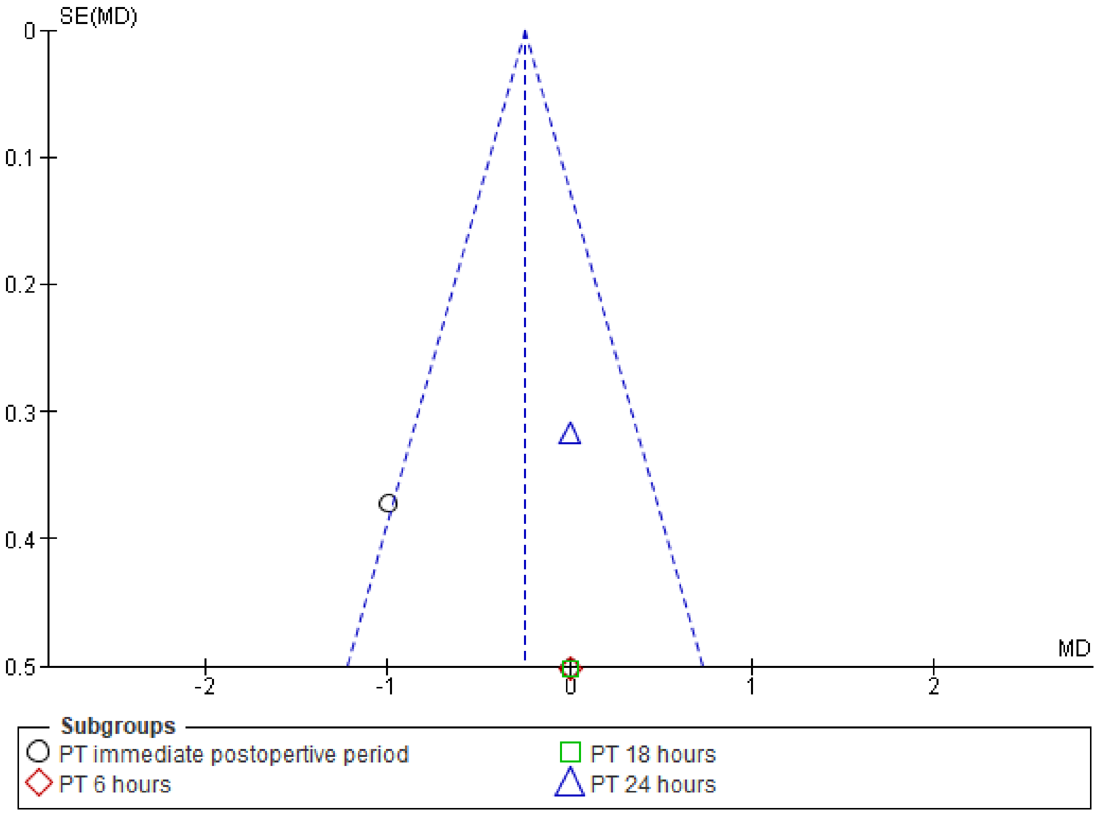
SE(MD)
0
0.1
0.2
0.3
0.4
0.5
-2
-1
0
1
2
MD
Subgroups
PT immediate postopertive period
PT 6 hours
PT 18 hours
PT 24 hours

H

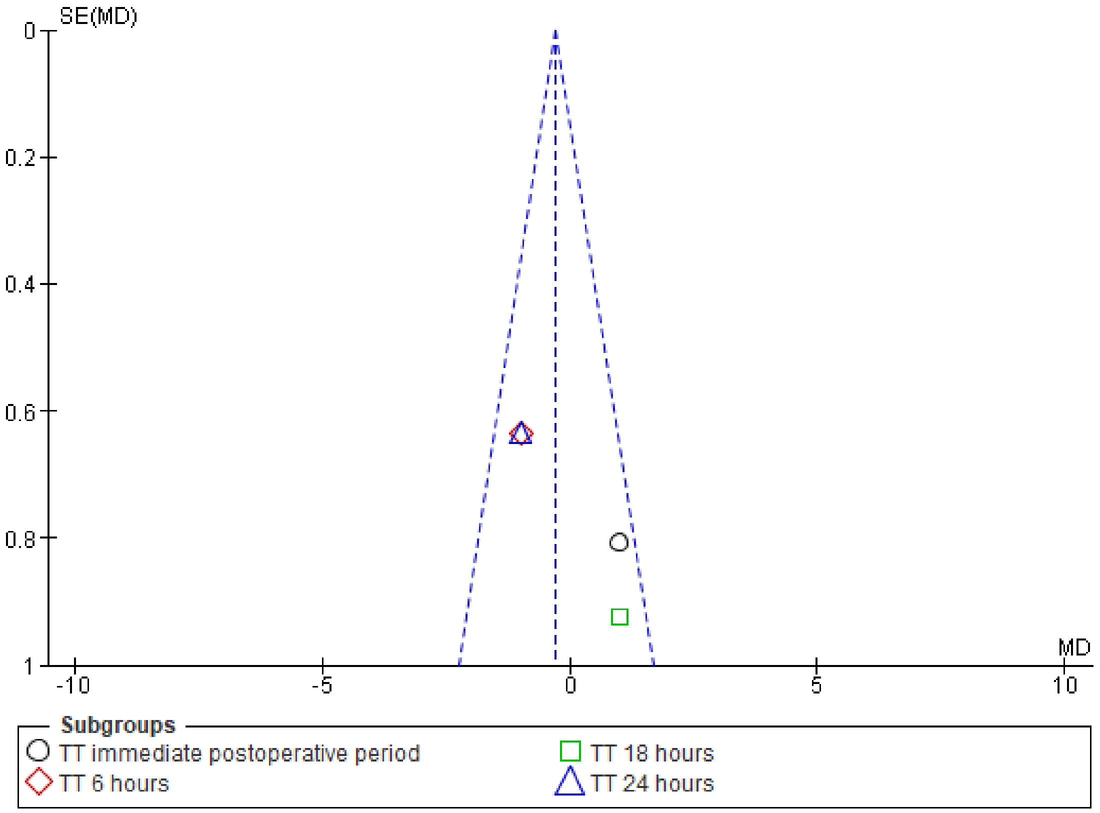
SE(MD)
0
0.2
0.4
0.6
0.8
1
-10
-5
0
5
10
MD
Subgroups
TT immediate postoperative period
TT 6 hours
TT 18 hours
TT 24 hours

I

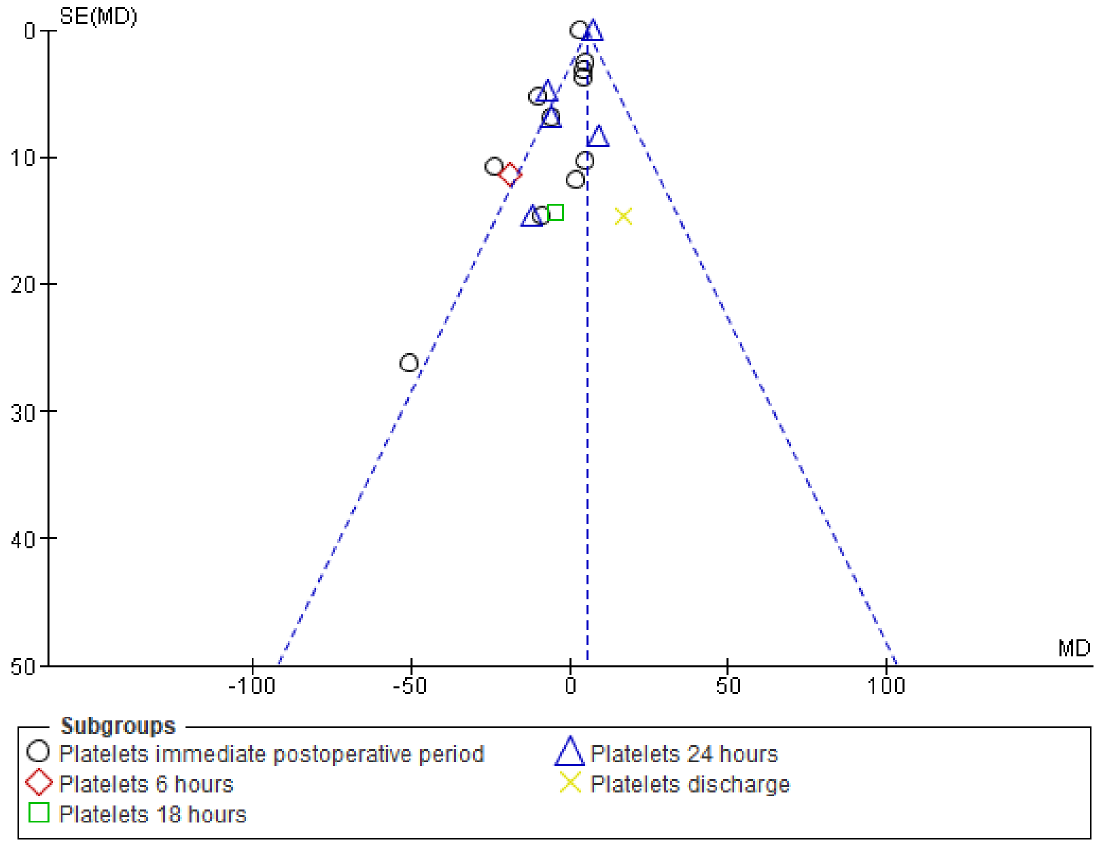
SE(MD)
0
10
20
30
40
50
-100
-50
0
50
100
MD
Subgroups
Platelets immediate postoperative period
Platelets 6 hours
Platelets 18 hours
Platelets 24 hours
Platelets discharge

J

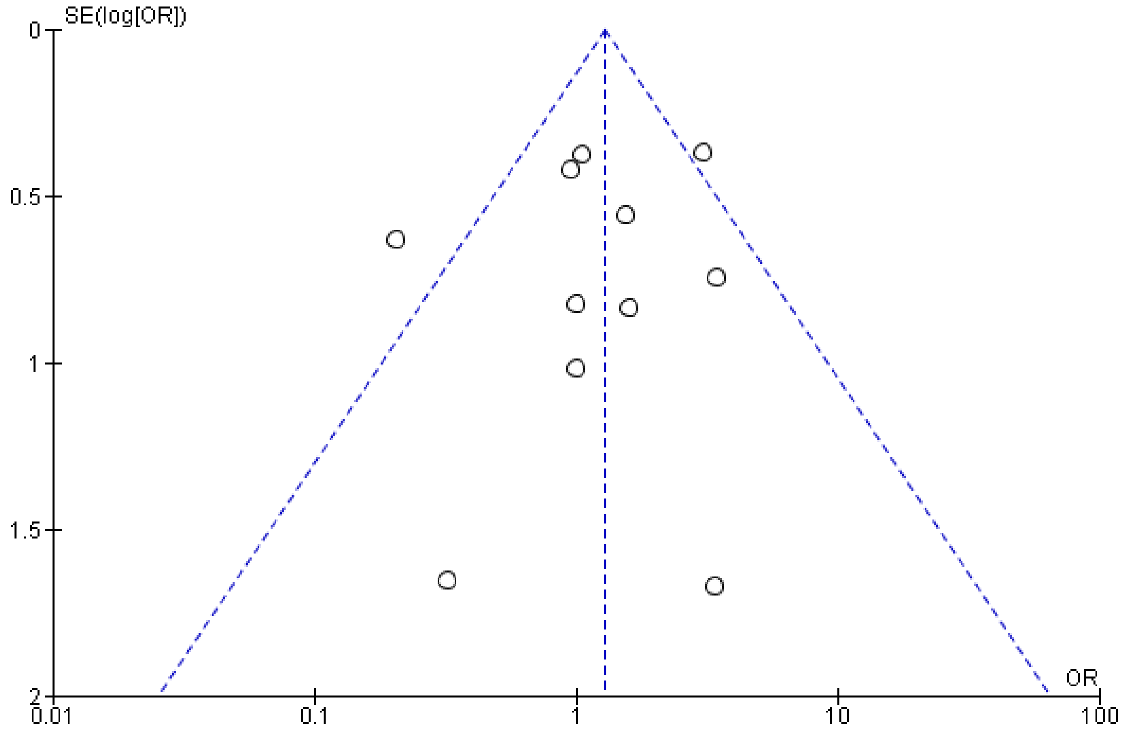
SE(log[OR])
0
0.5
1
1.5
2
0.01
0.1
1
10
100
OR

K

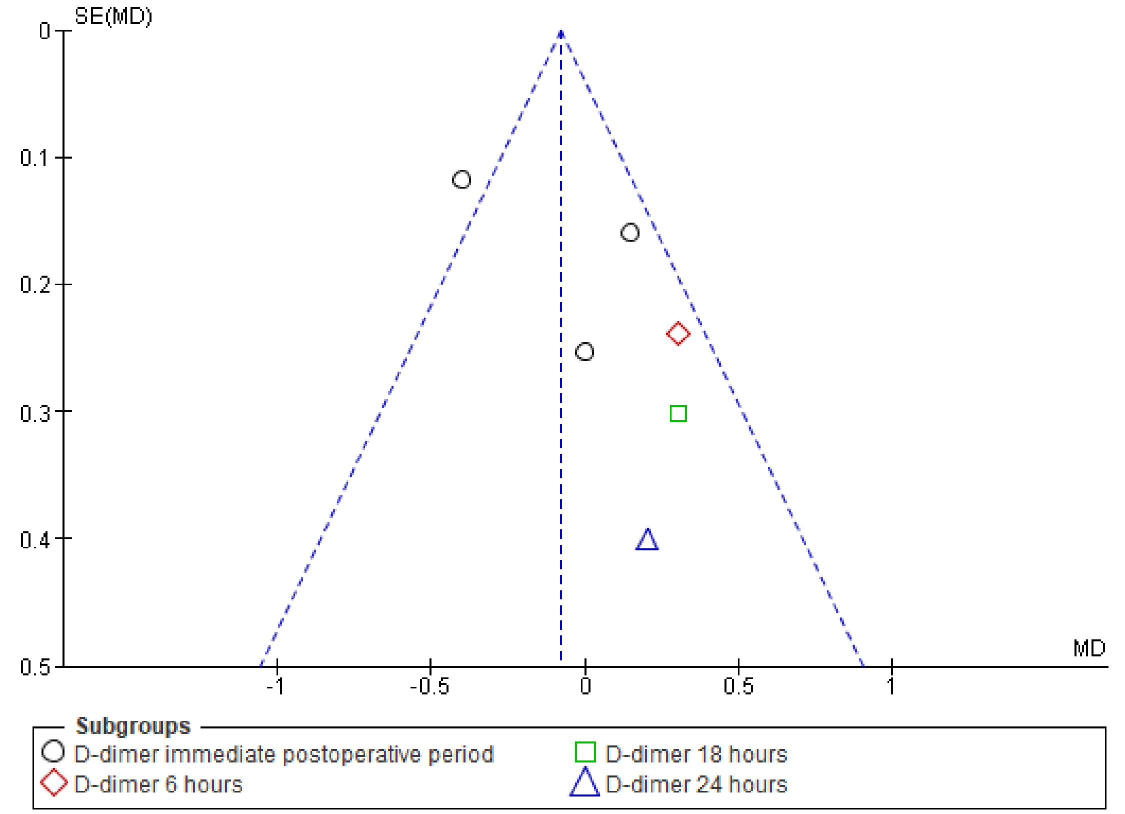
SE(MD)
0
0.1
0.2
0.3
0.4
0.5
MD
-1
-0.5
0
0.5
1
Subgroups
D-dimer immediate postoperative period
D-dimer 6 hours
D-dimer 18 hours
D-dimer 24 hours

L

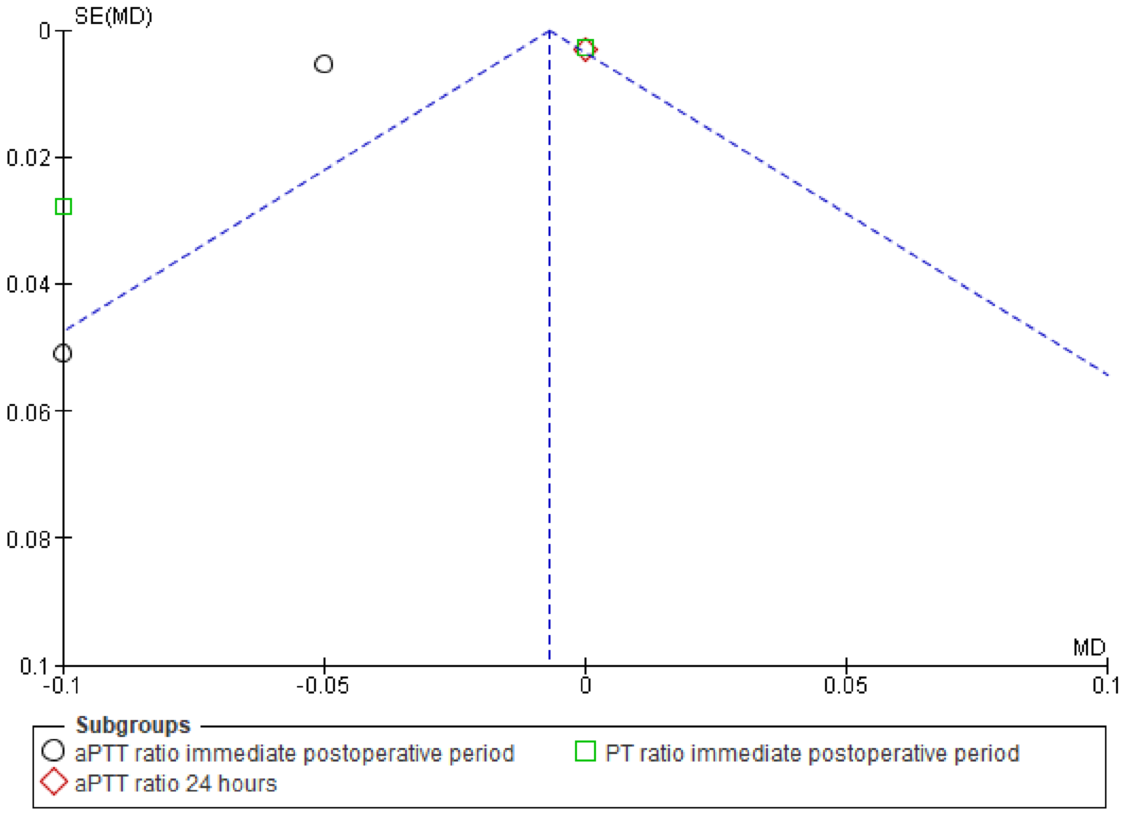
SE(MD)
0
0.02
0.04
0.06
0.08
0.1
MD
-0.1
-0.05
0
0.05
0.1
Subgroups
aPTT ratio immediate postoperative period
aPTT ratio 24 hours
PT ratio immediate postoperative period

(A): hemorrhage; (B): hemoglobin; (C): hematocrit; (D): fibrinogen; (E): INR; (F): aPTT (activated partial thromboplastin time) ; (G): PT (Protrombine time); (H): TT (Tromboplastine time); (I): platelets account; (J) FFP Fresh Frozen Plasma;(K): D-dimer; (L): aPTT ratio.
