# Supplementary material for: Evaluation of Red Blood Cell Biochemical Markers and Coagulation Profiles Following Cell Salvage in Cardiac Surgery: A Systematic Review and Meta-Analysis
Source: J Clin Med. 2024 Oct 11;13(20):6073. doi: 10.3390/jcm13206073 (PMC11508477; doi:10.3390/jcm13206073)
Supplement: Supplementary file 1 [file jcm-13-06073-s001.zip › Figures S4, S5 and S6.pdf]

Study or Subgroup	Cell Salvage Mean	Cell Salvage SD	Cell Salvage Total	Control Mean	Control SD	Control Total	Weight	Mean Difference IV, Random, 95% CI	A	B	C	D	E	F	G
**1.5.1 Fibrinogen inmmediate postoperative period**															
Boyle et al., 2019	0.8	0.1	26	0.8	0.1	28	21.7%	0.00 [-0.05, 0.05]	+	?	?	?	+	+	?
Daane et al. 2003	1.8	0.5	18	0.4	0.78	20	2.4%	1.40 [0.99, 1.81]	+	?	?	?	?	+	?
Murphy et al., 2005	2.21	0.034	30	2.34	0.033	31	24.6%	-0.13 [-0.15, -0.11]	+	+	+	?	?	+	?
Tachias et al., 2022	2.59	0.58	99	2.65	0.67	110	9.7%	-0.06 [-0.23, 0.11]	+	?	?	?	+	+	?
**Subtotal (95% CI)**			**173**			**189**	**58.5%**	**0.11 [-0.07, 0.29]**							
Heterogeneity: Tau² = 0.03; Chi² = 73.09, df = 3 (P < 0.00001); I² = 96%															
Test for overall effect: Z = 1.19 (P = 0.23)															
**1.5.2 Fibrinogen 6 hours**															
Daane et al. 2003	1.9	0.6	20	1.8	0.5	20	3.4%	0.10 [-0.24, 0.44]	+	?	?	?	?	+	?
**Subtotal (95% CI)**			**20**			**20**	**3.4%**	**0.10 [-0.24, 0.44]**							
Heterogeneity: Not applicable															
Test for overall effect: Z = 0.57 (P = 0.57)															
**1.5.3 Fibrinogen 18 hours**															
Daane et al. 2003	2.6	0.5	20	2.5	0.4	20	4.7%	0.10 [-0.18, 0.38]	+	?	?	?	?	+	?
**Subtotal (95% CI)**			**20**			**20**	**4.7%**	**0.10 [-0.18, 0.38]**							
Heterogeneity: Not applicable															
Test for overall effect: Z = 0.70 (P = 0.48)															
**1.5.4 Fibrinogen 24 hours**															
Daane et al. 2003	2.9	0.5	20	2.7	0.7	20	2.9%	0.20 [-0.18, 0.58]	+	?	?	?	?	+	?
Murphy et al., 2005	4.92	0.035	30	5.04	0.034	31	24.6%	-0.12 [-0.14, -0.10]	+	+	+	?	?	+	?
Tachias et al., 2022	4.62	0.81	99	4.54	0.98	110	5.9%	0.08 [-0.16, 0.32]	+	?	?	?	+	+	?
**Subtotal (95% CI)**			**149**			**161**	**33.4%**	**-0.01 [-0.20, 0.19]**							
Heterogeneity: Tau² = 0.02; Chi² = 5.34, df = 2 (P = 0.07); I² = 63%															
Test for overall effect: Z = 0.06 (P = 0.96)															
**Total (95% CI)**			**362**			**390**	**100.0%**	**-0.01 [-0.08, 0.05]**							
Heterogeneity: Tau² = 0.00; Chi² = 82.30, df = 8 (P < 0.00001); I² = 90%															
Test for overall effect: Z = 0.43 (P = 0.67)															
Test for subgroup differences: Chi² = 0.83, df = 3 (P = 0.84), I² = 0%															

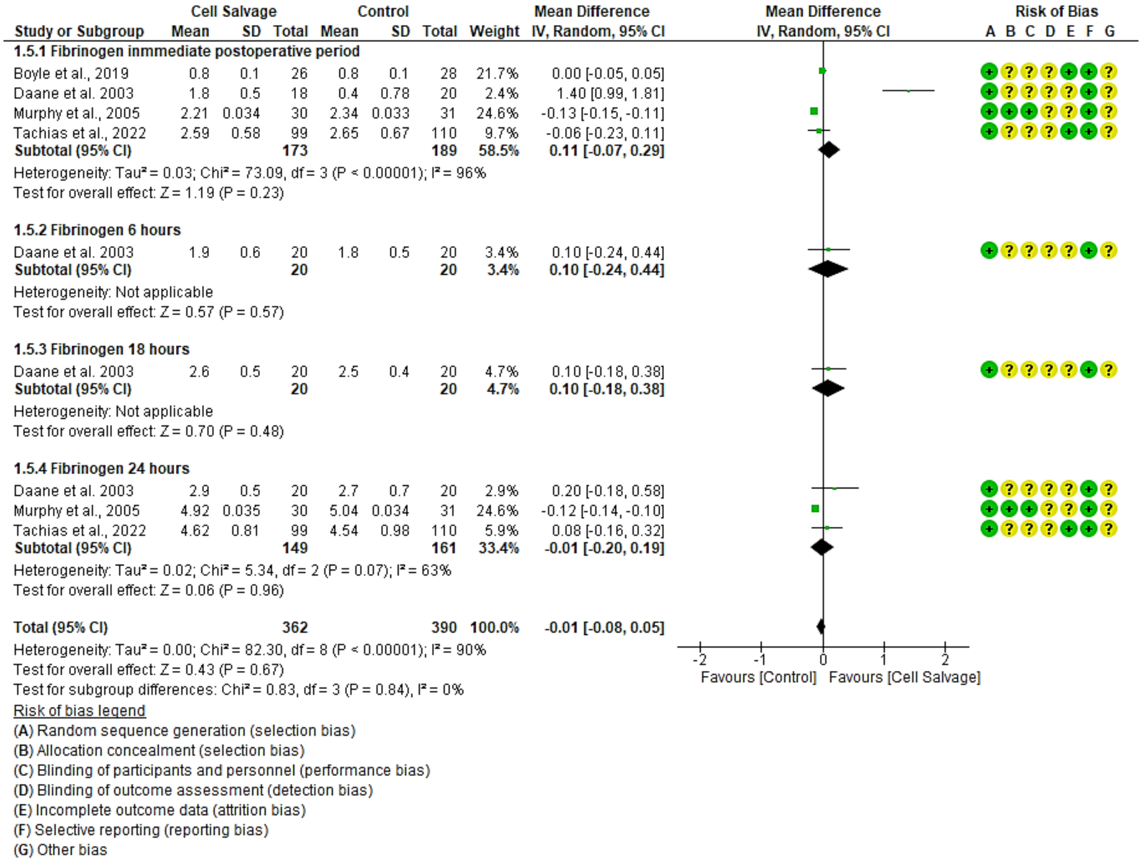

Risk of bias legend
(A) Random sequence generation (selection bias)
(B) Allocation concealment (selection bias)
(C) Blinding of participants and personnel (performance bias)
(D) Blinding of outcome assessment (detection bias)
(E) Incomplete outcome data (attrition bias)
(F) Selective reporting (reporting bias)
(G) Other bias

Figure S4: Forest plot representing fibrinogen over time.

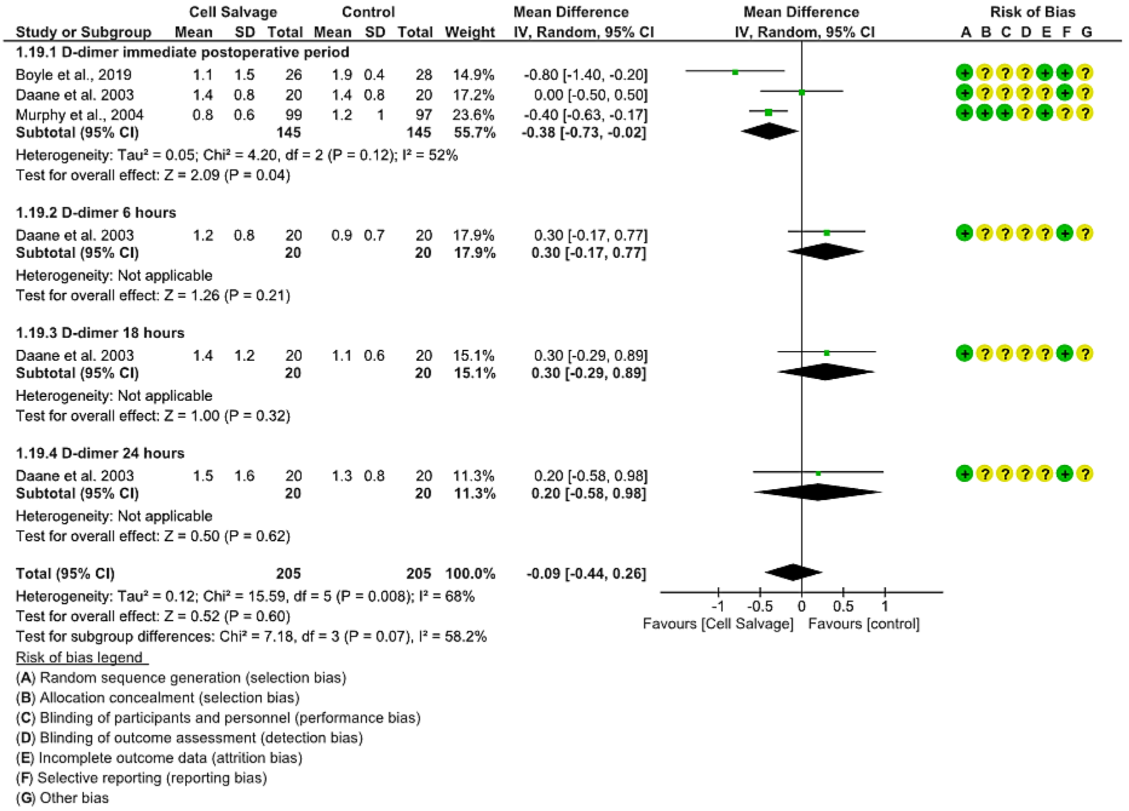

Figure S5: Forest plot representing D-dimer over time.

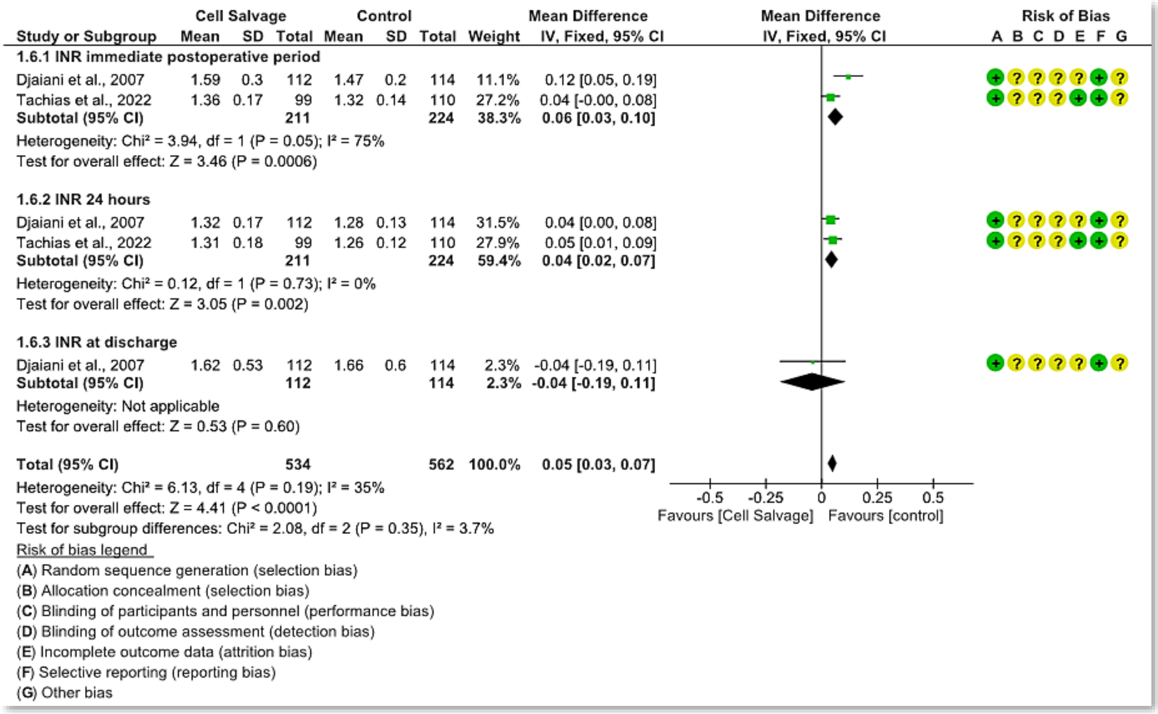

Figure S6: Forest plot representing INR through time.
