# Supplementary material for: Evaluation of Red Blood Cell Biochemical Markers and Coagulation Profiles Following Cell Salvage in Cardiac Surgery: A Systematic Review and Meta-Analysis
Source: J Clin Med. 2024 Oct 11;13(20):6073. doi: 10.3390/jcm13206073 (PMC11508477; doi:10.3390/jcm13206073)
Supplement: Supplementary file 1 [file jcm-13-06073-s001.zip › Table S1 GRADEPro.pdf]

Table S1: Grades of recommendations

Assessment of certainty							№ of patients		Effect		Certainty
Outcome	Nº of studies	Study design	Risk of bias	Inconsistency	Indirect evidence	Imprecision	Other considerations	Cell Salvage	Control Group	Relative (95% CI)	Absolute (95% CI)	
**Haemorrhage**	11	RCTs	Serious	Serious	It is not serious	It is not serious	publication bias is strongly suspected very strong association	1015	1041	-	MD **18.88** (-45.21 to 82.97)	⊕⊕⊕◯ Moderate
**Haemorrhage 6h**	4	RCTs	Very serious	Serious	It is not serious	It is not serious	publication bias is strongly suspected	160	159	-	MD **11.92** (-80.21 to 104.04)	⊕◯◯◯ Very low
**Hemorrhage 12 hours**	2	RCTs	Very serious	It is not serious	It is not serious	It is not serious	publication bias is strongly suspected n	288	274	-	MD **25.71** (-64.16 to 115.58)	⊕◯◯◯ Very low
**Hemorrhage 24 hours**	8	RCTs	Serious	Serious	It is not serious	It is not serious	publication bias is strongly suspected very strong association	438	480	-	MD **0.79** (-67.07 to 68.65)	⊕⊕◯◯ Low

Assessment of certainty							№ of patients		Effect		Certainty
Outcome	Nº of studies	Study design	Risk of bias	Inconsistency	Indirect evidence	Imprecision	Other considerations	Cell Salvage	Control Group	Relative (95% CI)	Absolute (95% CI)	
**Total Hemorrhage**	2	RCTs	Very serious	Very serious	It is not serious	It is not serious	publication bias is strongly suspected	129	128	-	MD **217.92 .** (- 489.53 to 925.37)	⊕◯◯◯ Very low
**Hemoglobin**	17	RCTs	Very serious	Serious	It is not serious	It is not serious	very strong association	1806	1865	-	MD **0.48** (0.28 to 0.69)	⊕⊕⊕◯ Moderate
**Hemoglobin immediate postoperative period**	13	RCTs	Very serious	It is not serious	It is not serious	It is not serious	strong association	665	686	-	MD **0.65** (0.27 to 1.04 .)	⊕⊕⊕◯ Moderate
**Hemoglobin 6 hours**	3	RCTs	Very serious	Very serious	It is not serious	It is not serious	publication bias is strongly suspected	149	152	-	MD **0.39** (-0.21 to 1.0)	⊕◯◯◯ Very low
**Hemoglobin 18 hours**	1	RCTs	Very serious	Very serious	Very serious	Very serious	publication bias is strongly suspected	20	20	-	MD **0.01** (-0.72 to 0.72)	⊕◯◯◯ Very low
**Hemoglobin 24 hours**	10	RCTs	Serious	Serious	It is not serious	It is not serious	strong association	667	702	-	MD **0.56** (0.23 to 0.9)	⊕⊕⊕◯ Moderate

Assessment of certainty							№ of patients		Effect		Certainty
Outcome	Nº of studies	Study design	Risk of bias	Inconsistency	Indirect evidence	Imprecision	Other considerations	Cell Salvage	Control Group	Relative (95% CI)	Absolute (95% CI)	
**Hemoglobin 48 hours**	1	RCTs	Very serious	Very serious	Very serious	Very serious	publication bias is strongly suspected	99	96	-	MD **0.5 .** (0.15 to 0.85)	⊕◯◯◯ Very low
**Hemoglobin discharge**	4	RCTs	Serious	It is not serious	It is not serious	It is not serious	publication bias is strongly suspected	206	209	-	MD **-0.05** (- 0.23 to 0.13)	⊕⊕◯◯ Low
**Hematocrit**	6	RCTs	Serious	It is not serious	It is not serious	It is not serious	publication bias is strongly suspected	311	355	-	MD **2.68** (1.04 to 4.32)	⊕⊕◯◯ Low
**Hematocrit immediate postoperative period**	5	RCTs	Serious	It is not serious	It is not serious	It is not serious	publication bias is strongly suspected	180	193	-	MD **3.91** (0.44 to 7.37)	⊕⊕◯◯ Low
**Hematocrit 6 hours**	1	RCTs	Very serious	Very serious	Very serious	Very serious	publication bias is strongly suspected	20	20	-	MD **1.0** (- 0.86 to 2.86)	⊕◯◯◯ Very low
**Hematocrit 18 hours**	1	RCTs	Very serious	Very serious	Very serious	Very serious	publication bias is strongly suspected	20	20	-	MD **1 .0** (-0.86 to 2.86)	⊕◯◯◯ Very low
**Hematocrit 24 hours**	3	RCTs	Serious	Serious	It is not serious	It is not serious	publication bias is strongly suspected	91	122	-	MD **2.33** (-0.07 to 4.74)	⊕◯◯◯ Very low

Assessment of certainty							№ of patients		Effect		Certainty
**Outcome**	**Nº of studies**	**Study design**	**Risk of bias**	**Inconsistency**	**Indirect evidence**	**Imprecision**	**Other considerations**	**Cell Salvage**	**Control Group**	**Relative (95% CI)**	**Absolute (95% CI)**	
**Fibrinogen**	4	RCTs	Serious	Serious	It is not serious	It is not serious	publication bias is strongly suspected	362	390	-	MD **-0.01** (-0.08 to 0.05)	⊕◯◯◯ Very low
**Fibrinogen inmmediate postoperative period**	4	RCTs	Serious	Serious	It is not serious	It is not serious	publication bias is strongly suspected	173	189	-	MD **0.11** (-0.07 to 0.29)	⊕◯◯◯ Very low
**Fibrinogen 6 hours**	1	RCTs	Very serious	Very serious	Very serious	Very serious	publication bias is strongly suspected	20	20	-	MD **0.1** (-0.24 to 0.44)	⊕◯◯◯ Very low
**Fibrinogen 18 hours**	1	RCTs	Very serious	Very serious	Very serious	Very serious	publication bias is strongly suspected	20	20	-	MD **0.1** (-0.18 to 0.38)	⊕◯◯◯ Very low
**Fibrinogen 24 hours**	3	RCTs	Serious	Serious	It is not serious	It is not serious	publication bias is strongly suspected	149	161	-	MD **-0.01** (-0.2 to 0.19)	⊕◯◯◯ Very low
**INR**	2	RCTs	Serious	Serious	It is not serious	It is not serious	publication bias is strongly suspected	534	562	-	MD **0.05** (0.03 to 0.07)	⊕◯◯◯ Very low
**INR immediate postoperative period**	2	RCTs	Serious	Serious	It is not serious	It is not serious	publication bias is strongly suspected	211	224	-	MD **0.06** (0.03 to 0.1 más alto.)	⊕◯◯◯ Very low

Assessment of certainty								№ of patients		Effect		Certainty
Outcome	Nº of studies	Study design	Risk of bias	Inconsistency	Indirect evidence	Imprecision	Other considerations	Cell Salvage	Control Group	Relative (95% CI)	Absolute (95% CI)	
**INR 24 hours**	2	RCTs	Serious	Serious	It is not serious	It is not serious	publication bias is strongly suspected	211	224	-	MD **0.04** (0.02 to 0.07)	⊕◯◯◯ Very low
**INR at discharge**	1	RCTs	Very serious	Very serious	Very serious	Very serious	publication bias is strongly suspected	112	114	-	MD **-0.04** (-0.19 to 0.11)	⊕◯◯◯ Very low
**aPTT**	5	RCTs	Serious	Serious	It is not serious	It is not serious	publication bias is strongly suspected	665	698	-	MD **0.5** (-2.5 to 3.49)	⊕◯◯◯ Very low
**aPTT immediate postoperative period**	5	RCTs	Serious	Serious	It is not serious	It is not serious	publication bias is strongly suspected	282	300	-	MD **0.87** (-5.01 to 6.76 .)	⊕◯◯◯ Very low
**aPTT 6 hours**	1	RCTs	Very serious	Very serious	Very serious	Very serious	publication bias is strongly suspected	20	20	-	MD **–2.0** (-5.1 to 1.1)	⊕◯◯◯ Very low
**aPTT 18 hours**	1	RCTs	Very serious	Very serious	Very serious	Very serious	publication bias is strongly suspected	20	20	-	MD **-1** (-3.48 to 1.48)	⊕◯◯◯ Very low
**aPTT 24 hours**	3	RCTs	Serious	Serious	It is not serious	It is not serious	publication bias is strongly suspected	231	244	-	MD **0.77** (-0.46 to 2.0.)	⊕◯◯◯ Very low

Assessment of certainty								№ of patients		Effect		Certainty
**Outcome**	**Nº of studies**	**Study design**	**Risk of bias**	**Inconsistency**	**Indirect evidence**	**Imprecision**	**Other considerations**	**Cell Salvage**	**Control Group**	**Relative (95% CI)**	**Absolute (95% CI)**	
**aPTT at discharge**	1	RCTs	Very serious	Very serious	Very serious	Very serious	publication bias is strongly suspected	112	114	-	MD **0.1** (-2.01 to 2.21)	⊕◯◯◯ Very low
**PT**	3	RCTs	Serious	Serious	It is not serious	It is not serious	publication bias is strongly suspected	131	136	-	MD **-0.44** (-1.04 to 0.17)	⊕◯◯◯ Very low
**PT immediate postopertive period**	3	RCTs	Serious	Serious	It is not serious	It is not serious	publication bias is strongly suspected	71	76	-	MD **-0.88** (- 1.48 to -0.29)	⊕◯◯◯ Very low
**PT 6 hours**	1	RCTs	Very serious	Very serious	Very serious	Very serious	publication bias is strongly suspected	20	20	-	MD .0.01 (-0.98 to 0.98.)	⊕◯◯◯ Very low
**PT 18 hours**	1	RCTs	Very serious	Very serious	Very serious	Very serious	publication bias is strongly suspected	20	20	-	MD **0.01** (-0.98 to 0.98)	⊕◯◯◯ Very low
**PT 24 hours**	1	RCTs	Very serious	Very serious	Very serious	Very serious	publication bias is strongly suspected	20	20	-	MD **0.01** (-0.62 to 0.62)	⊕◯◯◯ Very low
**TT**	1	RCTs	Very serious	Very serious	Very serious	Very serious	publication bias is strongly suspected	80	80	-	MD **-0.13** (-1.24 to 0.97)	⊕◯◯◯ Very low

Assessment of certainty								№ of patients		Effect		Certainty
Outcome	Nº of studies	Study design	Risk of bias	Inconsistency	Indirect evidence	Imprecision	Other considerations	Cell Salvage	Control Group	Relative (95% CI)	Absolute (95% CI)	
**TT immediate postoperative period**	1	RCTs	Very serious	Very serious	Very serious	Very serious	publication bias is strongly suspected	20	20	-	MD **1.0** (-0.58 to 2.58)	⊕◯◯◯ Very low
**TT 6 hours**	1	RCTs	Very serious	Very serious	Very serious	Very serious	publication bias is strongly suspected	20	20	-	MD **-1.0** (-2.24 to 0.24)	⊕◯◯◯ Very low
**TT 18 hours**	1	RCTs	Very serious	Very serious	Very serious	Very serious	publication bias is strongly suspected	20	20	-	MD **1.0** (-0.81 to 2.81)	⊕◯◯◯ Very low
**TT 24 hours**	1	RCTs	Very serious	Very serious	Very serious	Very serious	publication bias is strongly suspected	20	20	-	MD **–1.0** (-2.24 to 0.24)	⊕◯◯◯ Very low
**Platelets serie**	11	RCTs	Serious	Serious	It is not serious	It is not serious	publication bias is strongly suspected strong association	1062	1126	-	MD **1.48** (-1.28 to 4.25)	⊕⊕◯◯ Low
**Platelets immediate postoperative period**	10	RCTs	Serious	Serious	It is not serious	It is not serious	publication bias is strongly suspected strong association	608	626	-	MD - **0.24** (-4.71to 4.23)	⊕⊕◯◯ Low

Assessment of certainty							№ of patients		Effect		Certainty
Outcome	Nº of studies	Study design	Risk of bias	Inconsistency	Indirect evidence	Imprecision	Other considerations	Cell Salvage	Control Group	Relative (95% CI)	Absolute (95% CI)	
**Platelets 6 hours**	1	RCTs	Very serious	Very serious	Very serious	Very serious	publication bias is strongly suspected	20	20	-	MD **-19** (-41.32 to 3.32.)	⊕◯◯◯ Very low
**Platelets 18 hours**	1	RCTs	Very serious	Very serious	Very serious	Very serious	publication bias is strongly suspected	20	20	-	MD **-5** (-32.92 to 22.92)	⊕◯◯◯ Very low
**Platelets 24 hours**	5	RCTs	Serious	Serious	It is not serious	It is not serious	publication bias is strongly suspected	302	346	-	MD **0.11** (-8.76 to 8.98)	⊕◯◯◯ Very low
**Platelets discharge**	1	RCTs	Very serious	Very serious	Very serious	Very serious	publication bias is strongly suspected	112	114	-	MD **17** (-11.58 to 45.58)	⊕◯◯◯ Very low
**FFP**	11	RCTs	Serious	Serious	It is not serious	It is not serious	publication bias is strongly suspected<br>strong association	100/602 (16.6%)	72/551 (13.1%)	**OR 1.28** (0.92 a 1.78)	**31 per cent 1000** (from 9 minus to 80 plus)	⊕⊕◯◯ Low
**aPTT ratio**	2	RCTs	Serious	Very serious	It is not serious	It is not serious	publication bias is strongly suspected	288	287	-	MD **-0.03** (-0.05 to -0.01)	⊕◯◯◯ Very low

Assessment of certainty							№ of patients		Effect		Certainty
Outcome	Nº of studies	Study design	Risk of bias	Inconsistency	Indirect evidence	Imprecision	Other considerations	Cell Salvage	Control Group	Relative (95% CI)	Absolute (95% CI)	
**aPTT ratio immediate postoperative period**	2	RCTs	Serious	Very serious	It is not serious	It is not serious	publication bias is strongly suspected	129	128	-	MD **-0.05** (-0.06 to -0.04)	⊕◯◯◯ Very low
**aPTT ratio 24 hours**	1	RCTs	Very serious	Very serious	Very serious	Very serious	publication bias is strongly suspected	30	31	-	MD **0** (-0.01 to 0.01)	⊕◯◯◯ Very low
**PT ratio immediate postoperative period**	2	RCTs	Very serious	Very serious	It is not serious	It is not serious	publication bias is strongly suspected	129	128	-	MD **-0.05** (-0.14 to 0.05)	⊕◯◯◯ Very low
**D-dimer**	3	RCTs	Serious	Serious	It is not serious	It is not serious	publication bias is strongly suspected	205	205	-	MD **-0.09** (-0.44 to 0.26)	⊕◯◯◯ Very low
**D-dimer immediate postoperative period**	3	RCTs	Serious	It is not serious	It is not serious	It is not serious	publication bias is strongly suspected	145	145	-	MD **-0.38** (-0.73 to -0.02)	⊕⊕◯◯ Low
**D-dimer 6 hours**	1	RCTs	Very serious	Very serious	Very serious	Very serious	publication bias is strongly suspected	20	20	-	MD **0.3** (-0.17 to 0.77)	⊕◯◯◯ Very low

Assessment of certainty							№ of patients		Effect		Certainty
Outcome	Nº of studies	Study design	Risk of bias	Inconsistency	Indirect evidence	Imprecision	Other considerations	Cell Salvage	Control Group	Relative (95% CI)	Absolute (95% CI)	
**D-dimer 18 hours**	1	RCTs	Very serious	Very serious	Very serious	Very serious	publication bias is strongly suspected	20	20	-	MD **0.3** (-0.29 to 0.89)	⊕◯◯◯ Very low
**D-dimer 24 hours**	1	RCTs	Very serious	Very serious	Very serious	Very serious	publication bias is strongly suspected	20	20	-	MD **0.2**(-0.58 to 0.98)	⊕◯◯◯ Very low

The risk in the intervention group (and its 95% confidence interval) is based on the risk assumed in the comparison group and the relative effect of the intervention (and its 95% confidence interval). CI: Confidence Interval; RR: Risk Ratio. MD: Mean Difference. GRADE Working Group grades of evidence. High certainty: We are very confident that the true effect lies close to that of the effect estimate. Moderate certainty: We are moderately confident in the effect estimate—the true effect is likely to be close to the effect estimate, but there is a possibility that it is substantially different. Low certainty: Our confidence in the effect estimate is limited—the true effect may be substantially different from the effect estimate. Very low certainty: We have very little confidence in the effect estimate—the true effect is likely to be substantially different from the effect estimate.CI = confidence interval; RCTs = randomized controlled trial; RR = relative risk; ⊕⊕⊕◯ = level of recommendation.
